# Páramo is the world's fastest evolving and coolest biodiversity hotspot

**DOI:** 10.3389/fgene.2013.00192

**Published:** 2013-10-09

**Authors:** Santiago Madriñán, Andrés J. Cortés, James E. Richardson

**Affiliations:** ^1^Laboratorio de Botánica y Sistemática, Departamento de Ciencias Biológicas, Universidad de los Andes, BogotáDC, Colombia; ^2^Evolutionary Biology Centre, Department of Plant Ecology and Genetics, Uppsala UniversityUppsala, Sweden; ^3^Tropical Diversity Section, Royal Botanic Garden EdinburghEdinburgh, UK

**Keywords:** biodiversity hotspots, biogeography, evolutionary radiation, dated molecular phylogenies, net diversification rates, plant evolution, Páramos

## Abstract

Understanding the processes that cause speciation is a key aim of evolutionary biology. Lineages or biomes that exhibit recent and rapid diversification are ideal model systems for determining these processes. Species rich biomes reported to be of relatively recent origin, i.e., since the beginning of the Miocene, include Mediterranean ecosystems such as the California Floristic Province, oceanic islands such as the Hawaiian archipelago and the Neotropical high elevation ecosystem of the Páramos. Páramos constitute grasslands above the forest tree-line (at elevations of *c*. 2800–4700 m) with high species endemism. Organisms that occupy this ecosystem are a likely product of unique adaptations to an extreme environment that evolved during the last three to five million years when the Andes reached an altitude that was capable of sustaining this type of vegetation. We compared net diversification rates of lineages in fast evolving biomes using 73 dated molecular phylogenies. Based on our sample, we demonstrate that average net diversification rates of Páramo plant lineages are faster than those of other reportedly fast evolving hotspots and that the faster evolving lineages are more likely to be found in Páramos than the other hotspots. Páramos therefore represent the ideal model system for studying diversification processes. Most of the speciation events that we observed in the Páramos (144 out of 177) occurred during the Pleistocene possibly due to the effects of species range contraction and expansion that may have resulted from the well-documented climatic changes during that period. Understanding these effects will assist with efforts to determine how future climatic changes will impact plant populations.

“No zone of alpine vegetation in the temperate or cold parts of the globe can well be compared with that of the Páramos in the tropical Andes.” “Nowhere, perhaps, can be found collected together, in so small a space, productions so beautiful, and so remarkable in regard to the geography of plants.”

Alexander von Humboldt

*Aspects of Nature & Personal narrative*

## Introduction

The processes by which lineages diverge into new species are still poorly understood but are more likely to be determined in lineages that have recently speciated or are undergoing incipient speciation (Rieseberg and Willis, 2007). Biomes that have numerous examples of lineages that have speciated recently and rapidly would therefore be ideal places to study evolutionary phenomena. Studies that utilize dated phylogenies have reported high net diversification rates in a variety of biomes many of which are also designated biodiversity hotspots (Myers et al., 2000), for example Succulent Karoo (Klak et al., 2004) or the Mediterranean Basin (Valente et al., 2010). These radiations may have been caused by a variety of factors including recent geological activity (e.g., Hawaii that is part of the Polynesia-Micronesia hotspot) (Baldwin and Sanderson, 1998; Price and Wagner, 2004), or recent climatic change (e.g., Succulent Karoo).

**Figure 1:**
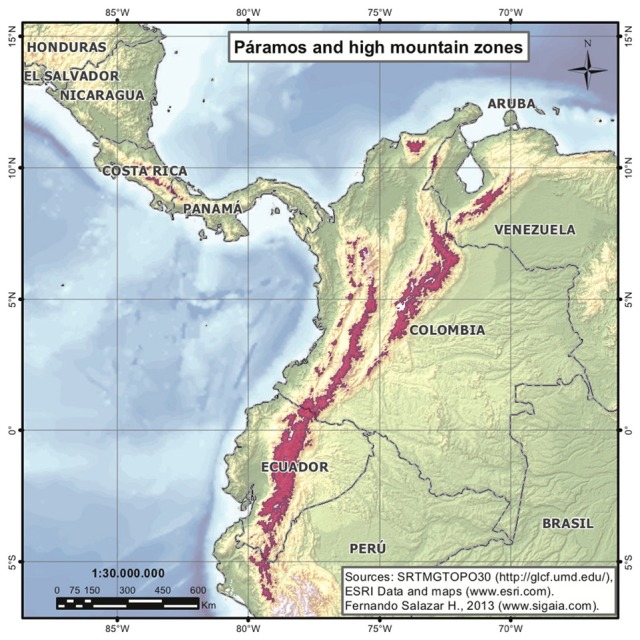
**Map indicating the present day area covered by Páramos (light red)**.

**Figure 2:**
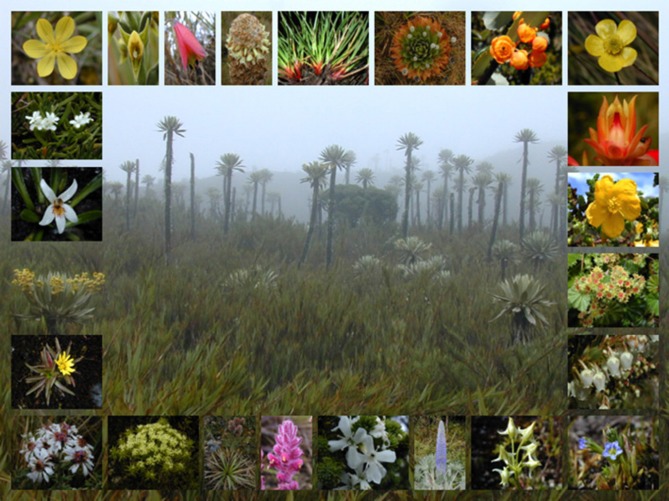
**Plants of the Páramos.** Center: Páramo landscape with *Chusquea tessellata* (foreground) and *Espeletia uribei* (background): Frame (clockwise from top left): *Sisyrinchium convolutum*, *Pterichis habenarioides*, *Bomarea pauciflora*, *Puya trianae*, *Oreobolus goeppingeri*, *Paepalanthus alpinus*, *Berberis goudotii*, *Ranunculus peruvianus*, *Echeveria bicolor*, *Hypericum goyanesii*, *Lachemilla orbiculata*, *Gaultheria anastomosans*, *Gentiana sedifolia*, *Halenia major*, *Lupinus alopecuroides*, *Aragoa abietina*, *Bartsia laniflora*, *Eryngium humboldtii*, *Myrrhidendron glaucescens*, *Diplostephium phylicoides*, *Hypochoeris sessiliflora*, *Espeletia killipii*, *Lysipomia laciniata* and *Valeriana stenophylla*.

In the Neotropics, lowland forests such as the Amazon have received a substantial amount of attention as species rich ecosystems (Hoorn et al., 2010). However, the high elevation tropical Andean Páramo ecosystem is not as widely recognized as a center of plant diversity. With 3431 species of vascular plants (Luteyn, 1999), Páramos may be considered a hotspot within a hotspot, as it is located within that of the Tropical Andes (Myers et al., 2000). Páramos are found at a number of isolated mountaintops at altitudes of between 2800 and 4700 m above sea level forming an archipelago-like distribution between latitudes of 11°N and 8°S covering approximately 35,000 km^2^ (Figure 1). The physical characteristics of the area occupied by this ecosystem include aseasonal conditions with high daytime and low nighttime temperatures, continuously high solar energy input, and high ultraviolet radiation (Luteyn, 1999). The great majority of the plant species found in the Páramos are endemic to this ecosystem, with close relatives in lowland-tropical or north- and south-temperate regions (van der Hammen and Cleef, 1986) (Figure 2). These ecosystems may be considered the “water towers” of South America as they provide large reservoirs that serve many of the major Andean cities. Páramos are under threat from mining activities (gold, coal, and lime) and climate change.

Evolution of the Páramo ecosystem was entirely dependent on the Andean orogeny as the ecosystem could only have developed once the Andes had reached a sufficient height. It has been estimated that the northern Andes reached 40% of its modern elevation from the mid-Miocene/early Pliocene and that they rose to current heights through rapid final uplift only by around 2.7 million years (Ma) ago (Gregory-Wodzicki, 2000; Mora et al., 2010). The northern Andes reached the altitude of the modern tree line that marks the lower limit of Páramo vegetation near the end of the Pliocene at 2.588 Ma ago (van der Hammen and Hooghiemstra, 2000). These are therefore the approximate dates by which conditions suitable for development of the Páramo ecosystem had established. By the late Pliocene/Early Pleistocene a proto-páramo vegetation occupied large areas between 2000 and 3000 m (van der Hammen, 1974; Hooghiemstra and van der Hammen, 2004). This vegetation type was characterized by pollen of modern Páramo elements such as Poaceae, *Valeriana*, *Plantago*, *Aragoa*, Ranunculaceae, Caryophyllaceae, *Geranium*, *Gunnera*, *Gentianella* and *Lysipomia*. However, pollen is not sufficiently diagnostic at species level to determine when speciation in these groups occurred.

Pleistocene changes in the distribution of this vegetation type and thus the species that occupy it are evident in the fossil record (van der Hammen, 1974). Individual plant species may have been forced to migrate vertically and the composition and distributions of plant communities would thus have been highly dynamic, with vegetation belts alternately contracting and expanding. During glacial maxima the area of Páramos was considerably larger than in inter-glacial periods as Páramo islands occupied lower elevations and thus merged when temperatures were lower (Hooghiemstra et al., 2006). These changes in distribution, which are largely mediated by temperature fluctuation, are more likely to be greater in a dissected montane landscape where there are rapid changes in elevation across small distances. These abiotic conditions would seem to be an ideal scenario for rapid allopatric speciation and perhaps also permit more rapid occupation of newly available and novel niche space. Indeed the Páramos have been characterized by several examples of rapid diversification events, demonstrated using dated molecular phylogenies (Särkinen et al., 2012), in genera such as *Gentianella* (von Hagen and Kadereit, 2001), *Valeriana* (Bell and Donoghue, 2005), *Lupinus* (Hughes and Eastwood, 2006) and *Hypericum* (Nürk et al., 2013). An increasing amount of sequence data and calibration points are becoming available permitting the production of dated phylogenies of plant groups from multiple lineages, allowing a comparison of net diversification rates amongst hotspots. Here we demonstrate that Páramos are undergoing an explosive phase of diversification that is both more rapid and more recent than in any other hotspot.

## Materials and methods

### Data and sampling for Páramo lineages

Sequence data generated in the laboratory of the first author or downloaded from GenBank were assembled and aligned for eight Páramo genera. Five additional phylogenies, taken from the literature, of genera containing Páramo clades were also included in this study (see Supporting Information). Dated phylogenies were estimated using the software package BEAST 1.4.8 (Drummond and Rambaut, 2007) using primary or secondary fossil calibrations or in one instance a geological calibration (see below and Table S1). In instances where these approaches to calibration were not possible we chose not to apply rates from other studies of taxa with a similar generation time (generation time has been shown to have an effect on rates) (Richardson et al., 2001; Smith and Donoghue, 2008) because of the expected elevated mutation rate, resulting from high U.V. light, in high altitude tropical ecosystems. Age estimates of crown nodes with confidence intervals were then utilized to estimate net diversification rates. Species and GenBank numbers for sequences used in the study are given in Appendix S1.

### Ages of clades from other hotspots

We compiled data from published dated phylogenetic studies from Páramo and other hotspots. These used a number of approaches to date phylogenies and we preferred those results that used internal fossil primary or secondary calibrations although in their absence those that used geological calibrations (i.e., oceanic island emergence) were considered acceptable. We also reported results of studies in hotspots other than Páramo that used borrowed rates from lineages with similar generation times but did not include these in our calculations for Páramo studies because, as mentioned above, we consider species that occupy that ecosystem to have an elevated substitution rate as a result of the intense U.V. light that is found in tropical highlands. This elevated rate might skew the result in favor of older age estimates of Páramo lineages. Lineages such as *Halenia* (Gentianaceae) should therefore actually have a higher net diversification rate than we would estimate by applying the fastest reported rate for herbaceous annuals.

If alternative options were available the date chosen was the one that was calibrated using fossils rather than geological events due to problems with the latter approach highlighted by Renner (2005). As different dates result from different analytical approaches we favored dates calculated by Bayesian methods followed by penalized likelihood and then NPRS (the latter has been shown to over-estimate ages) (Lavin et al., 2005). Favoring of Bayesian age estimates also permitted a more direct comparison with results of our analyses of Páramo lineages all of which used that approach.

Species numbers of *Heliophila* in each hotspot were taken from Marais (1970). The age reported for *Kokia* is that of the stem node and therefore an underestimate of the rate presented. Mediterranean studies of *Geranium* and *Erodium* are possible underestimates as only endemic species were included but those studies also included species outside the Mediterranean basin that we were unable to exclude because of a lack of distribution information. In some cases it was difficult to assess actual numbers of species of lineages in other biomes, e.g., Fabaceae lineages in the Cape Floristic Region likely have species that occur outside of that region. In some of our examples we included all species in a genus in our estimates even when it is likely that not all species are found within that biome which means we are overestimating net diversification rates in those lineages.

### Determination of ages of Páramo clades

A Bayesian dating method with a relaxed molecular clock was implemented using the program BEAST 1.4.8 (Drummond and Rambaut, 2007) to estimate divergence times. An XML (eXtensible Mark-up Language) input file was generated in the Bayesian Evolutionary Analysis Utility software (BEAUti) version v.1.4.8 (Drummond and Rambaut, 2007) (XML files for each analysis are available upon request to the corresponding author). The best performing evolutionary model was identified under two different model selection criteria, the hierarchical likelihood ratio test (hLRT) and the Akaike information criterion as implemented in MrModelTest (Nylander, 2004). Both selection criteria indicated that for each data set a General Time Reversible (GTR) with site heterogeneity being gamma distributed and with invariant sites model was optimal. An uncorrelated lognormal relaxed clock model was chosen based on the assumption of the absence of a molecular clock. To specify informative priors for all the parameters in the model, the Yule tree prior was used that was recommended as being appropriate for species-level phylogenies (Ho, 2007). As also recommended by Ho (2007), a log normal prior distribution was applied to fossil based calibrations and a normal distribution was used for secondary calibrations. The XML file was run in BEAST 1.4.8 (Drummond and Rambaut, 2007). Two runs were performed for each analysis. The MCMC chain length was set to 10,000,000, to screen every 10,000 and sample every 1000 trees. The resulting log file was imported into Tracer to check whether ESS values were adequate for each parameter. If they were not additional runs of 10,000,000 generations were performed until adequate ESS values were achieved. LogCombiner (Drummond and Rambaut, 2007) was used to combine tree files in cases where multiple runs were necessary. TreeAnnotator (Drummond and Rambaut, 2007) was used to produce the maximum clade credibility (MCC) tree that has the maximum sum of posterior probabilities on its internal nodes and summarizes the node height statistics in the posterior sample. MCC files were visualized using FigTree version 1.2.3 (Rambaut, 2009) and median and 95% highest posterior density (HPD) ages are reported in Table S1. We also calculated the number of species that, based on their median ages, diverged from their MRCA during the Pleistocene, i.e., within the last 2.58 million years, for each Páramo lineage.

### Calculation of net diversification rates

There are a number of diversification rate measures but we report that of the simple estimator of Kendall (1949) and Moran (1951) where *r* = ln(*N*) − ln(*N*_0_)]/*T* (where *N* = standing diversity, *N*_0_ = initial diversity, here taken as = 1, and *T* = inferred clade age). This estimate, a pure-birth model of diversification with a constant rate and no extinction, is the same as that of Magallón and Sanderson (2001).

### Statistical analyses

Average net diversification rates of all lineages within hotspots were calculated and a 95% bootstrap interval, using 1000 iterations, around each mean diversification rate was determined for each hotspot. Number of species, crown node age, mean diversification rate and number of Pleistocene speciation events for each Páramo lineage in the study are indicated in Table 1 (chronograms for each study are indicated in Figures S1A–H; Table S1 includes data on taxa from other hotspots).

**Table 1:** **Net diversification rates of Páramo plant lineages**.

					**Crown node age (Ma)**			**Net diversification Rate (*r*)**			
**Lineage**	**Family**	***n***	**Calibration node**	**Calibration age (Ma)**	**Minimum**	**Mean**	**Maximum**	**Minimum**	**Mean**	**Maximum**	**No. of Pleistocene speciations**
*Aragoa*	Plantaginaceae	17	Island *Plantago*	0.60	0.12	0.42	0.92	17.83	5.10	2.33	5 of 5
*Arcytophyllum*	Rubiaceae	14	*Arcytophyllum* stem node	21.50	6.48	10.96	16.36	0.30	0.18	0.12	1of 11
*Berberis*	Berberidaceae	32	Crown node of *Berberis*	37.30	0.07	3.80	9.7	39.61	0.73	0.29	4 of 17
*Calceolaria*	Calceolariaceae	65	*Jovellana*/*Calceolaria* split	15.00	1.42	2.50	3.51	2.45	1.39	0.99	23 of 23
*Draba*	Brassicaceae	55	Brassicaceae crown node	37.60	1.60	3.05	4.1	2.07	1.09	0.81	23 of 24
Espeletiinae	Asteraceae	120	*Barnadesia* split	45.70	2.42	4.04	5.92	1.69	1.01	0.69	21 of 22
*Festuca*	Poaceae	36	Loliinae crown node	13.80	1.87	4.28	7.66	1.55	0.68	0.38	3 of 5
*Jamesonia* + *Eriosorus*	Pteridaceae	32	Multiple fossils	7.60	n.a.	7.60	n.a.	n.a.	0.36	n.a.	0 of 2
*Lupinus*	Fabaceae	66	*Lupinus*/*Spartium* split	16.01	1.18	1.47	1.76	2.96	2.38	1.99	32 of 32
*Lysipomia*	Campanulaceae	27	*Lysipomia* crown node	11.10	6.61	8.96	11.19	0.39	0.29	0.23	13 of 20
*Oreobolus*	Cyperaceae	5	*O. furcatus*	5.10	1.67	3.01	4.71	0.55	0.30	0.19	3 of 5
*Puya*	Bromeliaceae	46	*Puya* stem node	9.10	0.26	0.80	1.58	12.06	3.92	1.98	10 of 10
*Valeriana*	Valerianaceae	53	Valerianaceae crown	55.00	9.46	14.58	19.69	0.35	0.22	0.17	n.a.

n, number of species within lineage; n.a., not available; Ma, million years ago.

Re-sampling without replacement was carried out 1000 times in order to estimate the probability that the fastest evolving lineage comes from a particular region. In each re-sampling step, five lineages per hotspot were randomly chosen across the eight hotspots, and the region where the fastest evolving lineage came from was identified from the 40 total randomly chosen lineages. The numbers of consecutive fastest evolving lineages that belong to the same region were also recorded. Two summary statistics per hotspot were calculated based on the 1000 sampling processes: the proportion of cases where the fastest evolving lineage came from a particular region and the maximum number of consecutive fastest evolving lineages that belong to the same region. Mean and confidence intervals for these two summary statistics were calculated running 1000 independent iterations of 1000 samples each. The summary statistics, their means and their confidence intervals are presented in Table S2.

## Results

**Figure 3:**
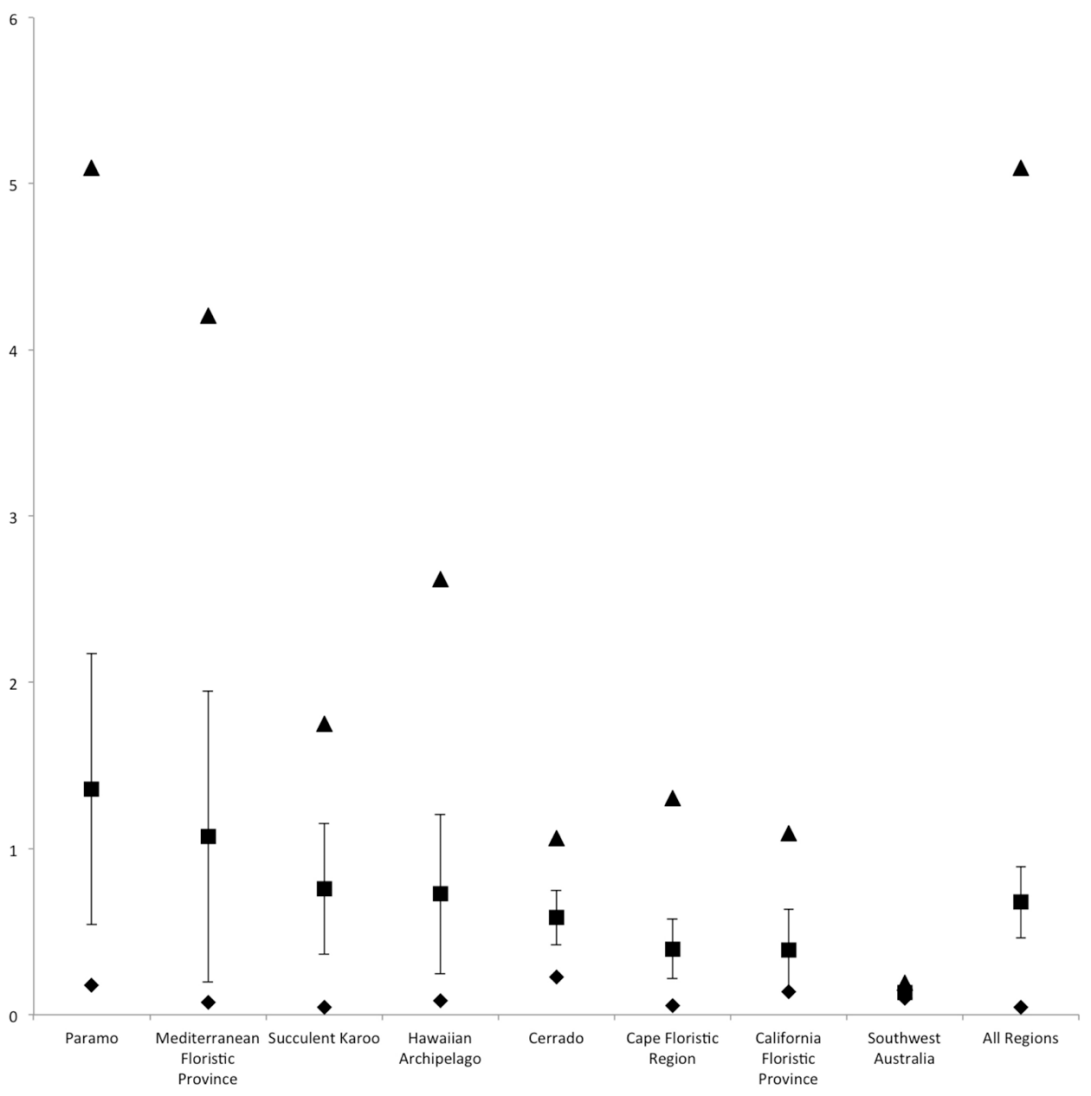
**Comparison of net diversification rates (*r*) in fast evolving biodiversity hotspots.** Upper (triangle), lower (diamond), mean (square) and Standard Deviation around the mean net diversification rates (Y axis) for each hotspot. Sample sizes: Páramos *n* = 13; Mediterranean Floristic Province *n* = 8; Succulent Karoo *n* = 9; Hawaiian Archipelago *n* = 9; Cerrado *n* = 10; Cape Floristic Region *n* = 13; California Floristic Province *n* = 6; Southwest Australia *n* = 5; All Regions *n* = 73.

Páramo lineages have higher net diversification rates than the fastest known lineages in other hotspots and have the fastest mean diversification rate of all hotspots (Figure 3; Table 2). The average diversification rate of the Páramo lineages sampled (Tables 1, 2) is 1.36 speciation events per million years (Myr-1; *n* = 13; we report values for a pure-birth model of diversification, one with a constant rate and no extinction *r* (Kendall, 1949; Moran, 1951; Magallón and Sanderson, 2001) (see Calculation of net diversification rates in methods above); rates factoring in extinction are reported in Table S1) compared with 1.07 Myr-1 in the Mediterranean Basin (*n* = 8), 0.76 Myr-1 in Succulent Karoo (*n* = 9), 0.73 Myr-1 in Hawaii (*n* = 9), 0.58 Myr-1 in Cerrado (*n* = 10), 0.40 Myr-1 in the Cape Floristic Region (*n* = 13), 0.39 Myr-1 in the California Floristic Province (*n* = 6) and 0.14 Myr-1 in Southwest Australia (*n* = 5). There are isolated lineages in some hotspots that have high rates such as in *Dianthus* (Valente et al., 2010) and core Ruschioideae (Klak et al., 2004). However, the average rate is significantly higher in Páramos than it is for a random sample of 13 from within our dataset of all hotspots. We also show that the fastest evolving lineage has a greater probability of being from the Páramos (0.51) than from any other hotspot (0.40 for the Mediterranean and 0.02 for Succulent Karoo; Table S2). The average number of fastest lineages that belong to the same region is also greatest in Páramos. In addition to the rapid net diversification rates, Páramos have a very high species density in comparison to other hotspots with 3431 species, nearly all of which occur nowhere else. Table 2 indicates the values for other hotspots and also that although the Cape Floristic Region has more species per kilometer squared than Páramos, the average rate per area in Páramos is greater than the Cape Floristic Region and all other hotspots studied.

**Table 2:** **Biodiversity Hotspots species richness and mean net diversification rates**.

**Region**	**Area**	**No. of**	**No. of**	**Mean Net**	**Speciation events per**
	**(km^2^)**	**species**	**species/km^2^**	**Diversification**	**million years per**
		**(endemics)**		**rate**	**km^2^**
California Floristic Province	293,804	8000 (2124)	0.027	0.39	1.32 × 10^−6^
Cape Floristic Region	78,555	9000 (6210)	0.196	0.40	5.05 × 10^−6^
Cerrado	2,031,990	12,669 (4215)	0.060	0.58	0.29 × 10^−6^
Hawaiian Archipelago	28,311	1004 (*c*. 900)	0.035	0.73	25.68 × 10^−6^
Mediterranean Floristic Province	2,085,292	22,500 (11,700)	0.010	1.07	0.52 × 10^−6^
Páramos	35,000	3431 (*)	0.098	1.36	38.80 × 10^−6^
Southwest Australia	356,717	5500 (2948)	0.015	0.14	0.38 × 10^−6^
Succulent Karoo	102,691	6350 (2439)	0.062	0.76	7.38 × 10^−6^

^*^Precise number of endemic species unknown, but close to 100%.

## Discussion

We demonstrate that Páramos not only have had a rapid diversification rate but those radiations have also been more recent than in other hotspots. In addition these diversifications have occurred more or less over the same period of time in multiple unrelated lineages in contrast to, for example, the single rapid diversification of cichlid fishes that occurred in a restricted area in East African lakes (Kornfield and Smith, 2000). Additional recent studies of evolutionary histories are also consistent with the rapid diversification of other Páramo lineages (Vargas and Madriñán, 2012; Nürk et al., 2013). Because Páramo lineages are in an early explosive phase of diversification we expect current species composition to be the result of on-going speciation processes with extinction having a minimal effect. Possible causes of diversification in Páramos include allopatric speciation resulting from distribution changes caused by climatic cycles during the Pleistocene or, adaptation to numerous new microclimates and substrates resulting from geological activity associated with Andean uplift. We acknowledge that diversification of some lineages may have occurred prior to the Plio-Pleistocene. However, our chronograms indicate that, based on median ages, 144 of the 176 Páramo species in our study split from their MRCA during the Pleistocene (Table 1) that is consistent with them having arisen as a result of inter-glacial range contractions in that epoch. We assume that the addition of more species to our sample will increase this figure. Although allopatric speciation could be the primary cause of isolation, the high levels of ultraviolet light are likely to induce a rapid mutation rate (Davies et al., 2004; Willis et al., 2009), and therefore hasten morphological differentiation and perhaps reproductive isolation with these mutations being more likely to become fixed in small fragmented populations. It is also possible that ecological opportunity and physiographic heterogeneity were the primary factors driving rapid diversification, e.g., Andean *Lupinus* (Hughes and Eastwood, 2006). The actual processes of speciation remain unclear, however, what is evident from this study is that it occurred more rapidly in Páramos than in any other hotspot on earth and confirms Hughes and Eastwood's (2006) prediction that the species-richness of the flora is the result of a set of rapid plant radiations.

Species that occupy steep altitudinal gradients are likely to undergo altitudinal range shifts with changes in temperature and are therefore ideal organisms to model the effects of historical and potential future changes. High altitude restricted species are also the most threatened due to the limited areas into which they can migrate under conditions of increasing temperatures such as those we are currently experiencing. The relatively small areas of Páramo vegetation make them logistically easier to study. For example, fragmented areas of Páramos around Bogotá that are as little as 30 km apart and would likely (based on palaeobotanical evidence from the Sabana de Bogotá (van der Hammen, 1973, 1974; van der Hammen and Cleef, 1986) have been connected during the last glacial maximum may be studied to look for signatures of fragmentation processes that occurred within the last 10,000 years and in previous inter-glacial periods.

When faced with changing climatic conditions, such as temperature increases, populations respond either by adapting, going extinct or migrating (Fordham et al., 2012). The contraction and expansion of populations that is very evident in Páramos according to palaeoecological data (van der Hammen, 1974; Hooghiemstra and van der Hammen, 2004), and that could have resulted in the high number of Pleistocene speciation events reported here, is indicative of an inability to adapt to changing conditions, as demonstrated in other montane systems (Colwell et al., 2008; Kelly and Goulden, 2008; Lenoir et al., 2008). This inability to adapt over periods of thousands of years reinforces the dangers that plant populations face in these environments when challenged by changes that might occur over shorter time scales of decades or centuries. Research into Páramo plants will help us to understand past and future evolutionary processes and provide the information necessary to help to conserve this and other ecosystems in the face of the continuing pressures exerted by anthropogenic climatic alterations.

### Conflict of interest statement

The authors declare that the research was conducted in the absence of any commercial or financial relationships that could be construed as a potential conflict of interest.
