# Supplementary material for: Páramo is the world's fastest evolving and coolest biodiversity hotspot
Source: Front Genet. 2013 Oct 9;4:192. doi: 10.3389/fgene.2013.00192 (PMC3793228; doi:10.3389/fgene.2013.00192)
Supplement: Supplementary file 1 [file DataSheet1.PDF]

SUPPLEMENTARY MATERIAL

Supporting figure caption

Figure S1 (a–h). Chronograms for each Páramo lineage with mean and 95% HPD's of node ages. Páramo lineages are indicated by boxes; calibration points are indicated with an asterisk (see Table S1 for references). **a.** *Aragoa*; **b.** *Arcytophyllum*; **c.** *Calceolaria*; **d.** *Draba*; **e.** Espeletiineae; **f.** *Festuca*; **g.** *Lysipomia*; **h.** *Puya*. All studies were based on ITS sequence data.

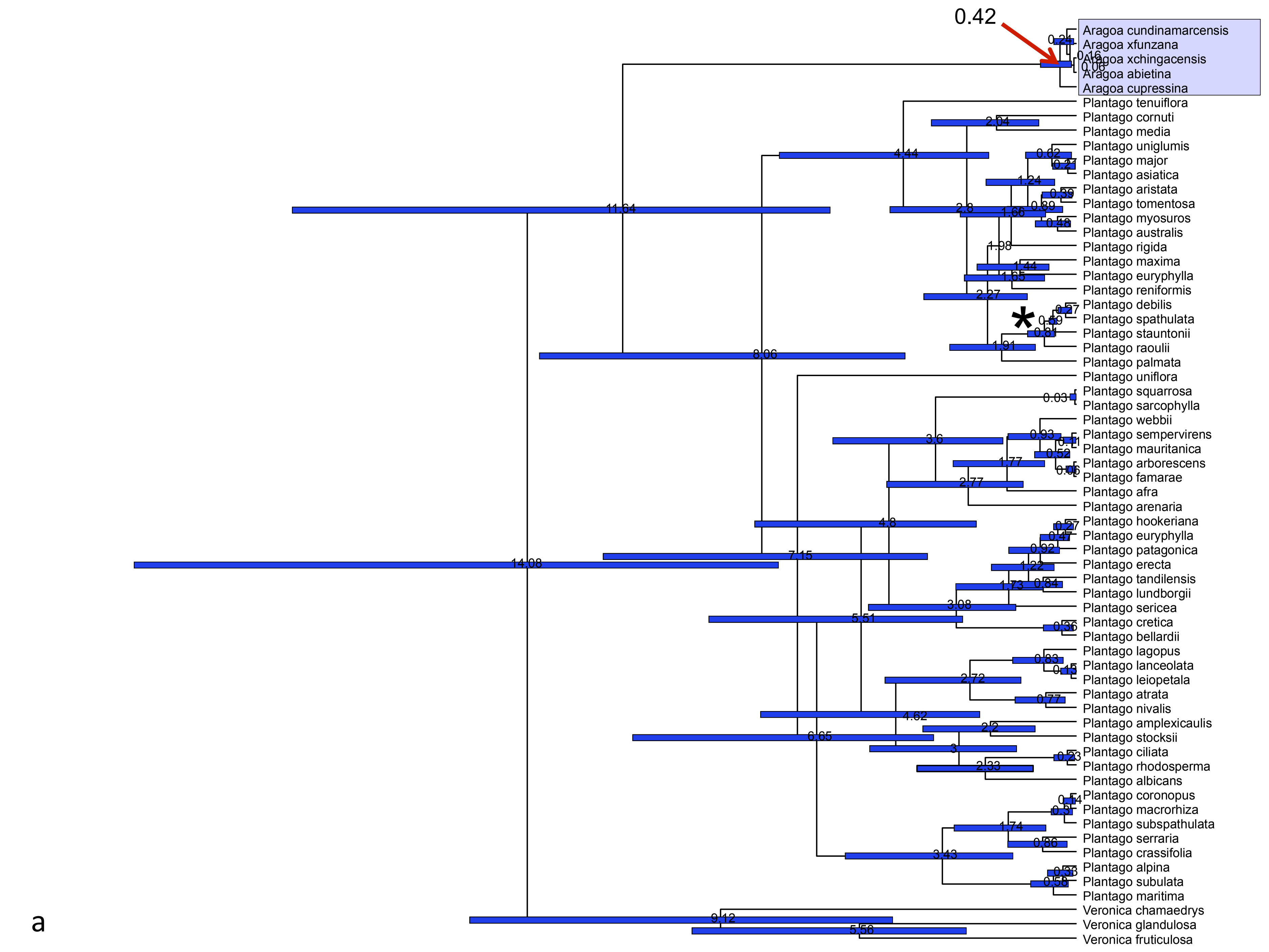
0.42
Aragoa cundinamarcensis
Aragoa xfunzana
Aragoa xchingacensis
Aragoa abietina
Aragoa cupressina
Plantago tenuiflora
Plantago cornuti
Plantago media
Plantago uniglumis
Plantago major
Plantago asiatica
Plantago aristata
Plantago tomentosa
Plantago myosuros
Plantago australis
Plantago rigida
Plantago maxima
Plantago euryphylla
Plantago reniformis
Plantago debilis
Plantago spathulata
Plantago stauntonii
Plantago raoulii
Plantago palmata
Plantago uniflora
Plantago squarrosa
Plantago sarcophylla
Plantago webbii
Plantago sempervirens
Plantago mauritanica
Plantago arborescens
Plantago famarae
Plantago afra
Plantago arenaria
Plantago hookeriana
Plantago euryphylla
Plantago patagonica
Plantago erecta
Plantago tandilensis
Plantago lundborgii
Plantago sericea
Plantago cretica
Plantago bellardii
Plantago lagopus
Plantago lanceolata
Plantago leiopetala
Plantago atrata
Plantago nivalis
Plantago amplexicaulis
Plantago stocksii
Plantago ciliata
Plantago rhodosperma
Plantago albicans
Plantago coronopus
Plantago macrorhiza
Plantago subspathulata
Plantago serraria
Plantago crassifolia
Plantago alpina
Plantago subulata
Plantago maritima
Veronica chamaedrys
Veronica glandulosa
Veronica fruticulosa
14.08
11.64
8.06
7.15
6.65
5.51
5.56
9.12
4.8
4.62
4.44
3.6
3.43
3.08
3
2.8
2.77
2.72
2.33
2.27
2.2
2.04
1.98
1.91
1.77
1.74
1.73
1.66
1.65
1.44
1.24
1.22
0.93
0.92
0.89
0.83
0.81
0.77
0.66
0.62
0.03
a

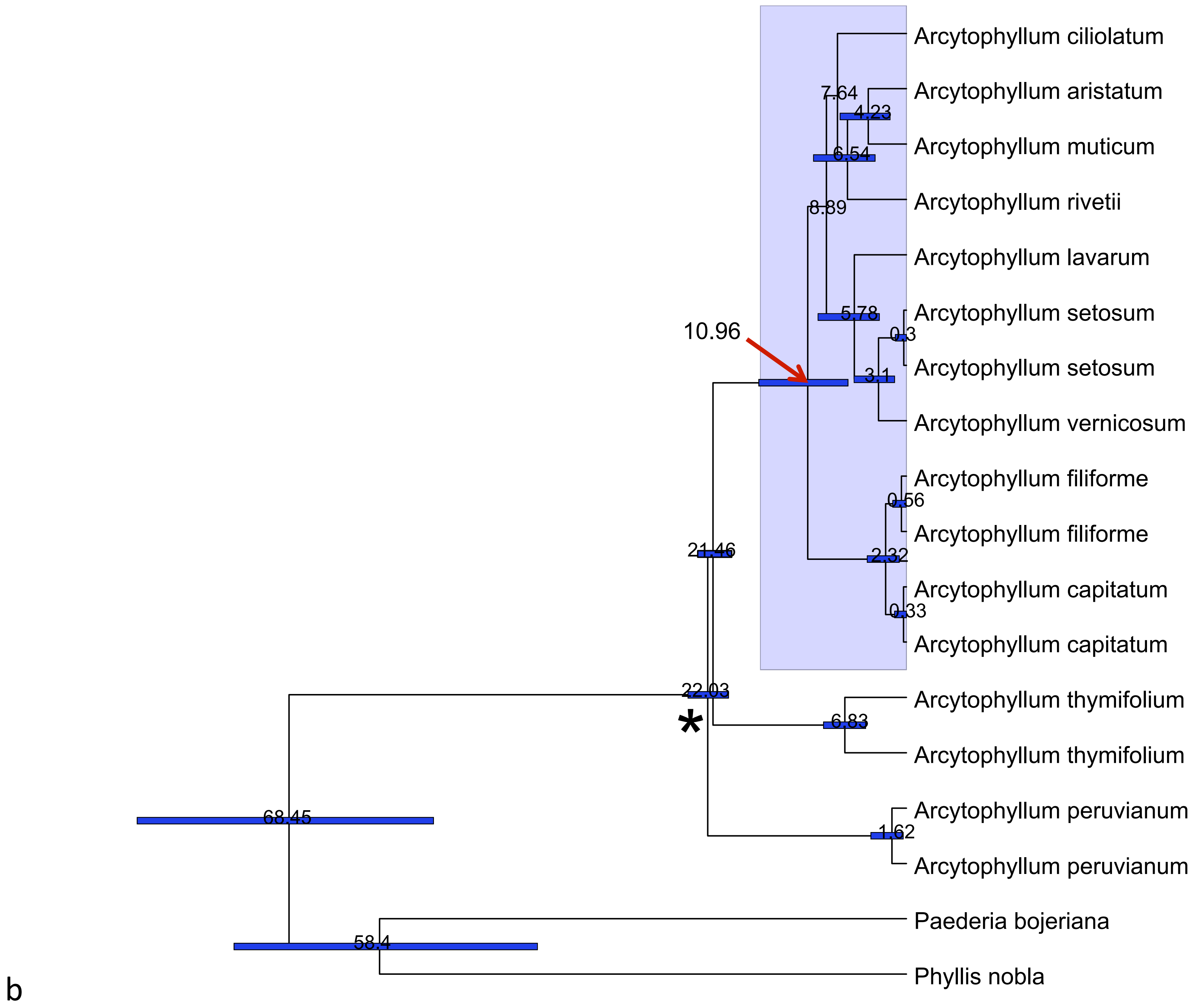
Arcytophyllum ciliolatum
Arcytophyllum aristatum
Arcytophyllum muticum
Arcytophyllum rivetii
Arcytophyllum lavarum
Arcytophyllum setosum
Arcytophyllum setosum
Arcytophyllum vernicosum
Arcytophyllum filiforme
Arcytophyllum filiforme
Arcytophyllum capitatum
Arcytophyllum capitatum
Arcytophyllum thymifolium
Arcytophyllum thymifolium
Arcytophyllum peruvianum
Arcytophyllum peruvianum
Paederia bojeriana
Phyllis nobla
10.96
7.64
4.23
6.54
8.89
5.78
0.3
3.1
0.56
2.32
0.33
21.46
22.03
*
6.83
1.62
68.45
58.4
b

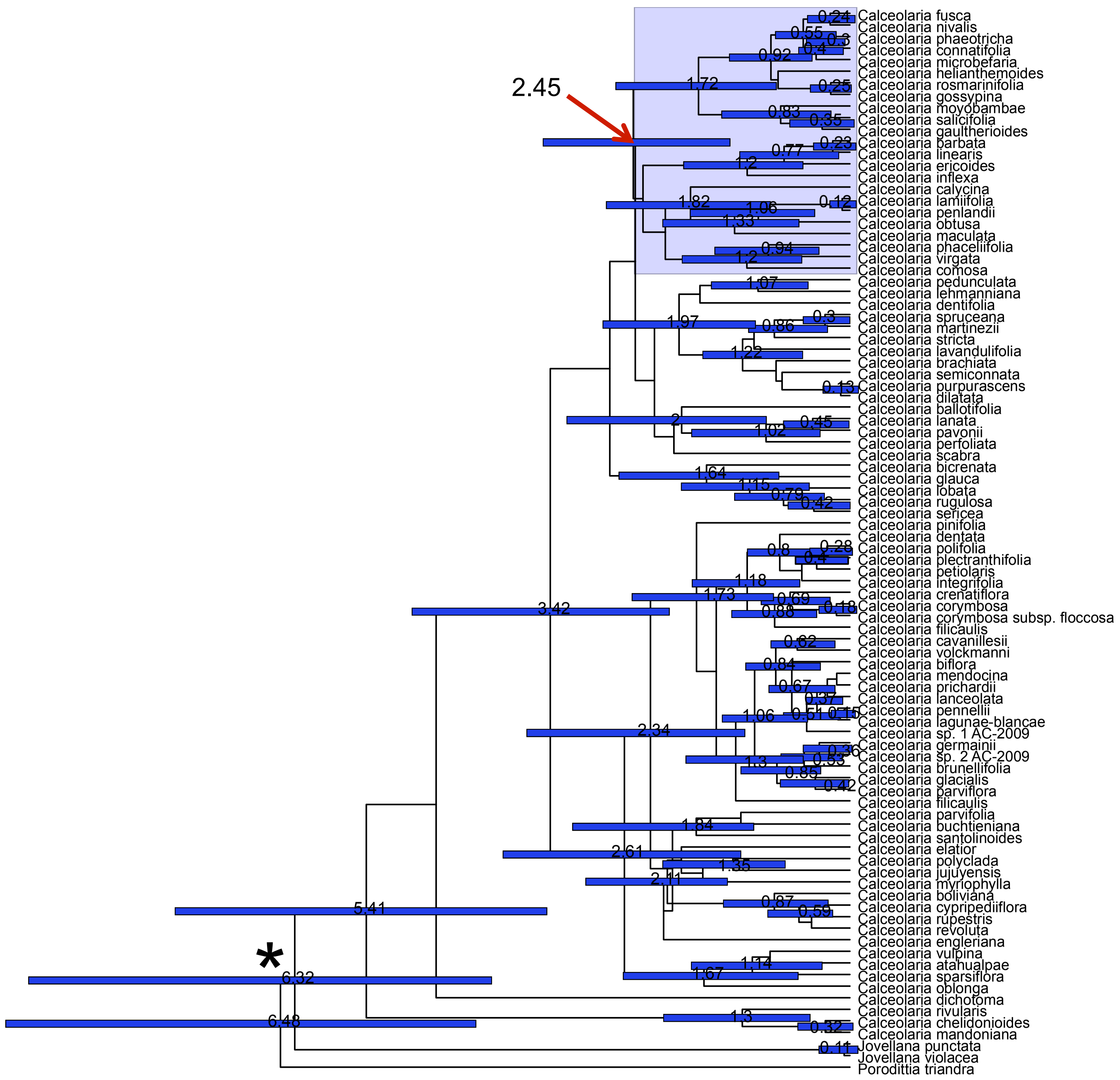
2.45
Calceolaria fusca
Calceolaria nivalis
Calceolaria phaeotricha
Calceolaria connatifolia
Calceolaria microbefaria
Calceolaria helianthemoides
Calceolaria rosmarinifolia
Calceolaria gossypina
Calceolaria moyobambae
Calceolaria salicifolia
Calceolaria gaultherioides
Calceolaria barbata
Calceolaria linearis
Calceolaria ericoides
Calceolaria inflexa
Calceolaria calycina
Calceolaria lamiifolia
Calceolaria penlandii
Calceolaria obtusa
Calceolaria maculata
Calceolaria phaceliifolia
Calceolaria virgata
Calceolaria comosa
Calceolaria pedunculata
Calceolaria lehmanniana
Calceolaria dentifolia
Calceolaria spruceana
Calceolaria martinezii
Calceolaria stricta
Calceolaria lavandulifolia
Calceolaria brachiata
Calceolaria semiconnata
Calceolaria purpurascens
Calceolaria dilatata
Calceolaria ballotifolia
Calceolaria lanata
Calceolaria pavonii
Calceolaria perfoliata
Calceolaria scabra
Calceolaria bicrenata
Calceolaria glauca
Calceolaria lobata
Calceolaria rugulosa
Calceolaria sericea
Calceolaria pinifolia
Calceolaria dentata
Calceolaria polifolia
Calceolaria plectranthifolia
Calceolaria petiolaris
Calceolaria integrifolia
Calceolaria crenatiflora
Calceolaria corymbosa
Calceolaria corymbosa subsp. floccosa
Calceolaria filicaulis
Calceolaria cavanillesii
Calceolaria volckmanni
Calceolaria biflora
Calceolaria mendocina
Calceolaria prichardii
Calceolaria lanceolata
Calceolaria pennellii
Calceolaria lagunae-blancae
Calceolaria sp. 1 AC-2009
Calceolaria germainii
Calceolaria sp. 2 AC-2009
Calceolaria brunellifolia
Calceolaria glacialis
Calceolaria parviflora
Calceolaria filicaulis
Calceolaria parvifolia
Calceolaria buchtieniana
Calceolaria santolinoides
Calceolaria elatior
Calceolaria polyclada
Calceolaria jujuyensis
Calceolaria myriophylla
Calceolaria boliviana
Calceolaria cypripediiflora
Calceolaria rupestris
Calceolaria revoluta
Calceolaria engleriana
Calceolaria vulpina
Calceolaria atahualpae
Calceolaria sparsiflora
Calceolaria oblonga
Calceolaria dichotoma
Calceolaria rivularis
Calceolaria chelidonioides
Calceolaria mandoniana
Jovellana punctata
Jovellana violacea
Porodittia triandra

c

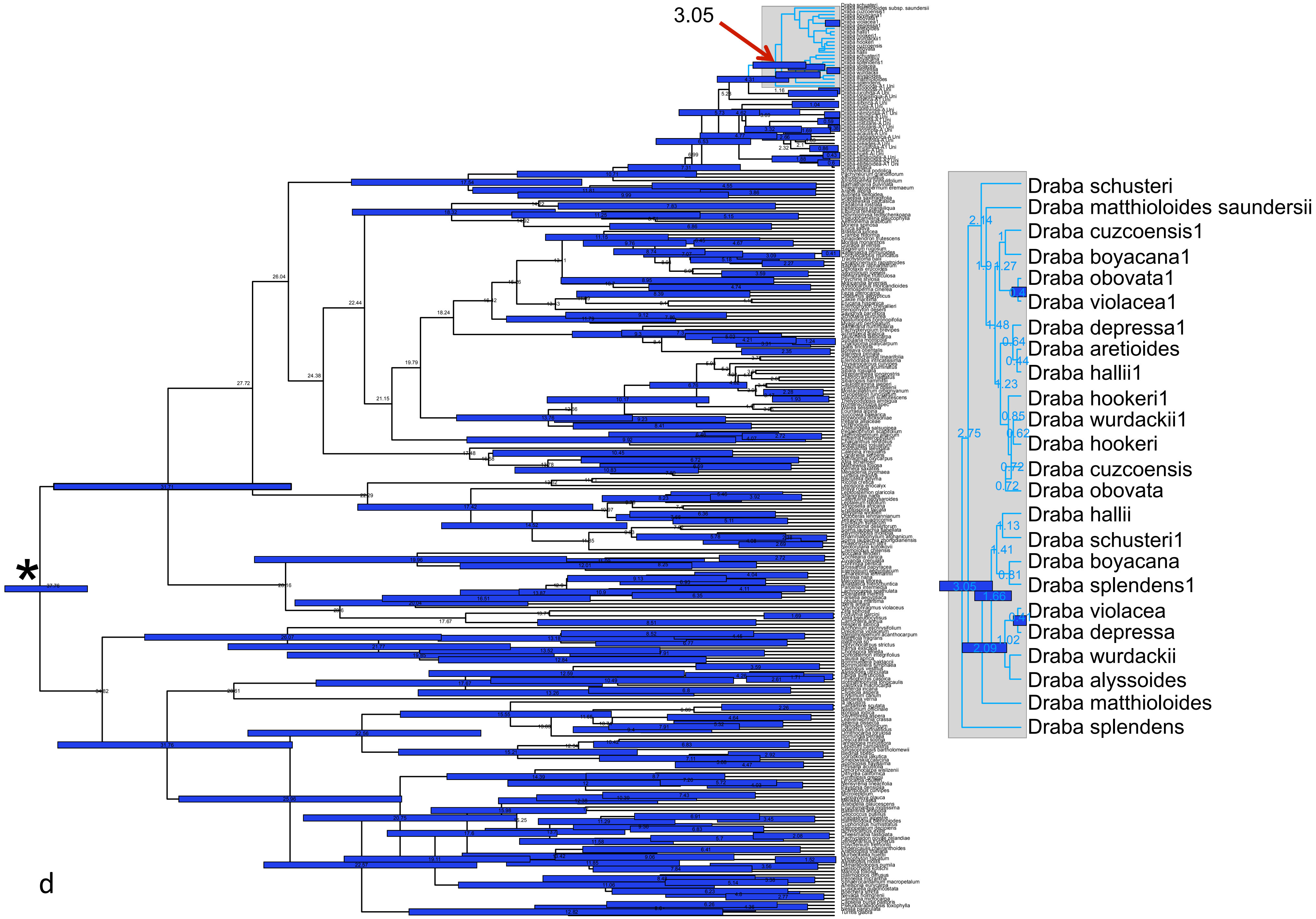
3.05
d
*
Draba schusteri
Draba matthioloides saundersii
Draba cuzcoensis1
Draba boyacana1
Draba obovata1
Draba violacea1
Draba depressa1
Draba aretioides
Draba hallii1
Draba hookeri1
Draba wurdackii1
Draba hookeri
Draba cuzcoensis
Draba obovata
Draba hallii
Draba schusteri1
Draba boyacana
Draba splendens1
Draba violacea
Draba depressa
Draba wurdackii
Draba alyssoides
Draba matthioloides
Draba splendens

e

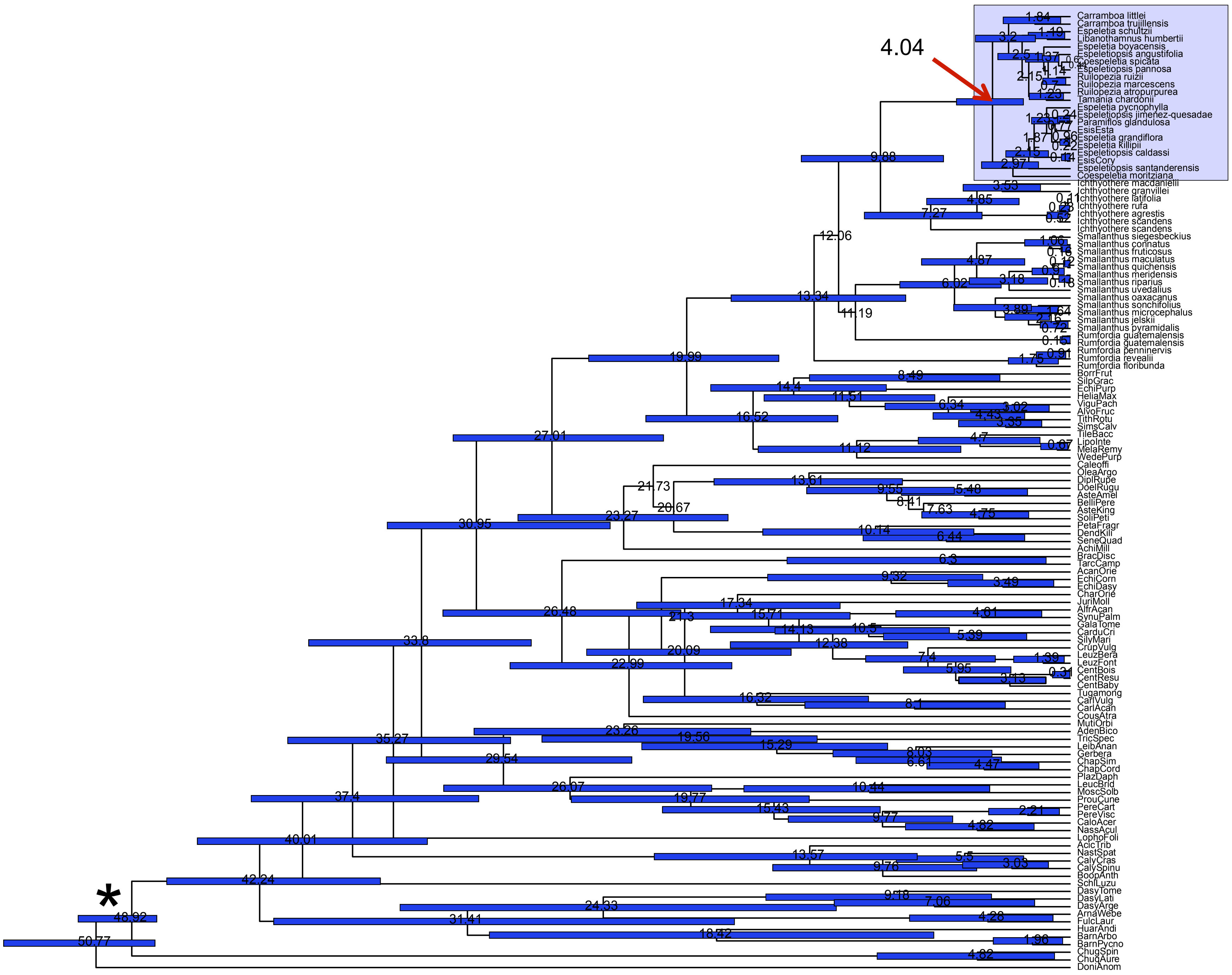
4.04
*
Carramboa littlei
Carramboa trujillensis
Espeletia schultzii
Libanothamnus humbertii
Espeletia boyacensis
Espeletiopsis angustifolia
Coespeletia spicata
Espeletiopsis pannosa
Ruilopezia ruizii
Ruilopezia marcescens
Ruilopezia atropurpurea
Tamania chardonii
Espeletia pycnophylla
Espeletiopsis jimenez-quesadae
Paramiflos glandulosa
EsisEsta
Espeletia grandiflora
Espeletia killipii
Espeletiopsis caldassi
EsisCory
Espeletiopsis santanderensis
Coespeletia moritziana
Ichthyothere macdanielii
Ichthyothere granvillei
Ichthyothere latifolia
Ichthyothere rufa
Ichthyothere agrestis
Ichthyothere scandens
Ichthyothere scandens
Smallanthus siegesbeckius
Smallanthus connatus
Smallanthus fruticosus
Smallanthus maculatus
Smallanthus quichensis
Smallanthus meridensis
Smallanthus riparius
Smallanthus uvedalius
Smallanthus oaxacanus
Smallanthus sonchifolius
Smallanthus microcephalus
Smallanthus jelskii
Smallanthus pyramidalis
Rumfordia guatemalensis
Rumfordia guatemalensis
Rumfordia penninervis
Rumfordia revealii
Rumfordia floribunda
BorrFrut
SilpGrac
EchiPurp
HeliaMax
ViguPach
AlvoFruc
TithRotu
SimsCalv
TileBacc
LipoInte
MelaRemy
WedePurp
Caleoffi
OleaArgo
DiplRupe
DoelRugu
AsteAmel
BelliPere
AsteKing
SoliPeti
PetaFragr
DendKili
SeneQuad
AchiMill
BracDisc
TarcCamp
AcanOrie
EchiCorn
EchiDasy
CharOrie
JuriMoll
AlfrAcan
SynuPalm
GalaTome
CarduCri
SilyMari
CrupVulg
LeuzBera
LeuzFont
CentBois
CentResu
CentBaby
Tugamong
CarlVulg
CarlAcan
CousAtra
MutiOrbi
AdenBico
TricSpec
LeibAnan
Gerbera
ChapSim
ChapCord
PlazDaph
LeucBrid
MoscSolb
ProuCune
PereCart
PereVisc
CaloAcer
NassAcul
LophoFoli
AcicTrib
NastSpat
CalyCras
CalySpinu
BoopAnth
SchlLuzu
DasyTome
DasyLati
DasyArge
ArnaWebe
FulcLaur
HuarAndi
BarnArbo
BarnPycno
ChugSpin
ChugAure
DoniAnom

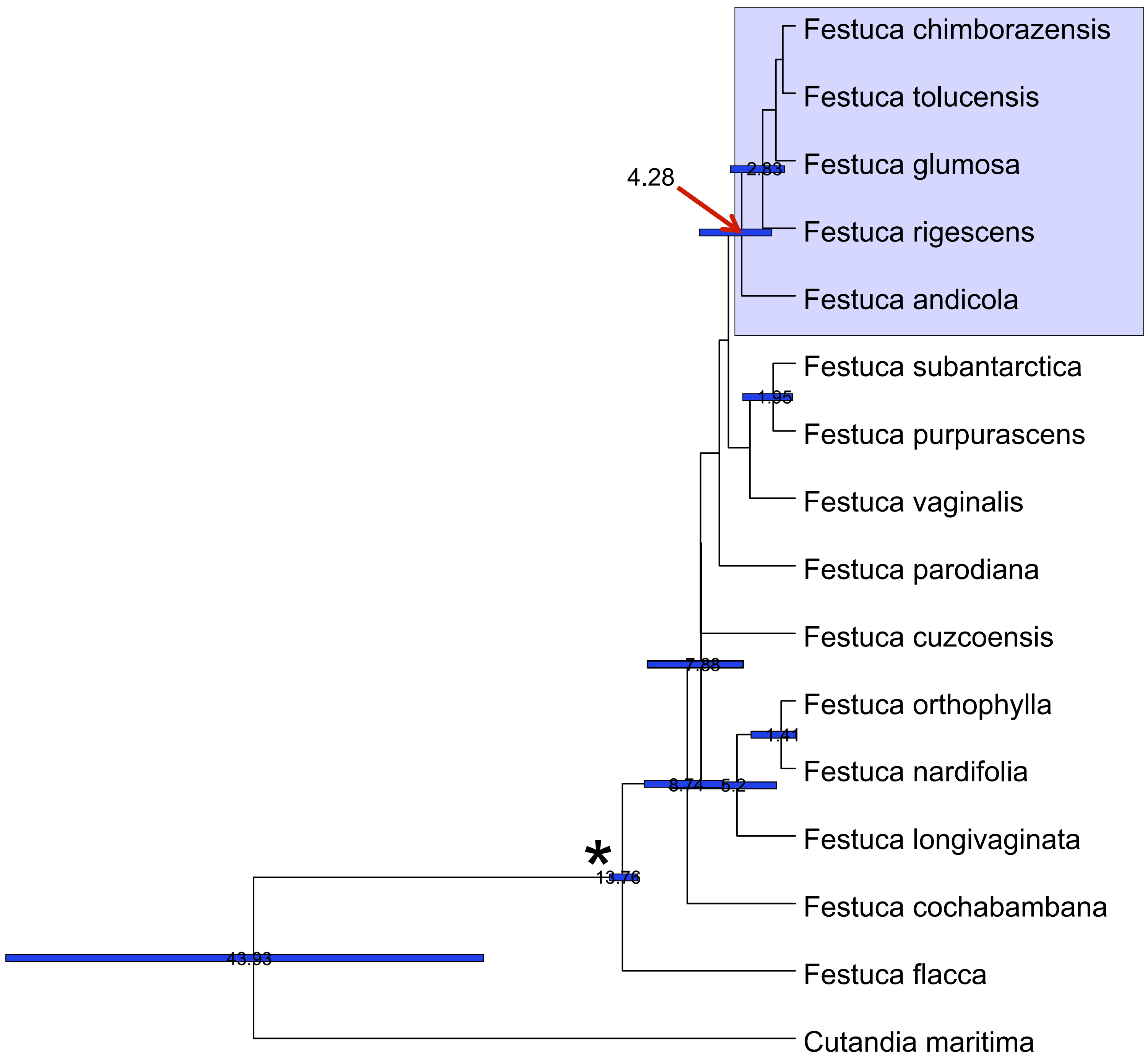

Festuca chimborazensis
Festuca tolucensis
Festuca glumosa
Festuca rigescens
Festuca andicola
Festuca subantarctica
Festuca purpurascens
Festuca vaginalis
Festuca parodiana
Festuca cuzcoensis
Festuca orthophylla
Festuca nardifolia
Festuca longivaginata
Festuca cochabambana
Festuca flacca
Cutandia maritima
4.28
2.83
1.95
7.88
1.41
8.74
5.2
13.76
43.93
*

f

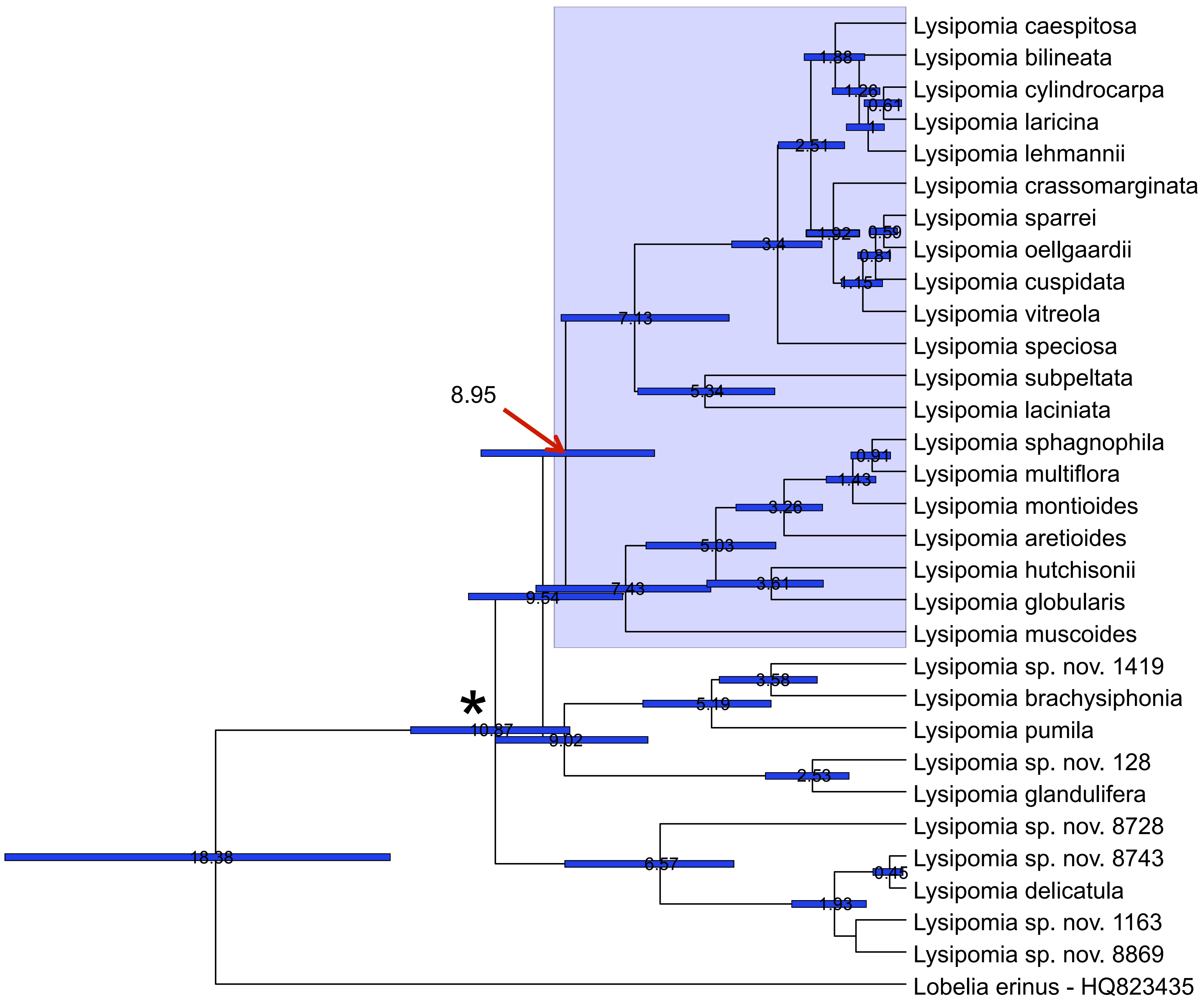
Lysipomia caespitosa
Lysipomia bilineata
Lysipomia cylindrocarpa
Lysipomia laricina
Lysipomia lehmannii
Lysipomia crassomarginata
Lysipomia sparrei
Lysipomia oellgaardii
Lysipomia cuspidata
Lysipomia vitreola
Lysipomia speciosa
Lysipomia subpeltata
Lysipomia laciniata
Lysipomia sphagnophila
Lysipomia multiflora
Lysipomia montioides
Lysipomia aretioides
Lysipomia hutchisonii
Lysipomia globularis
Lysipomia muscoides
Lysipomia sp. nov. 1419
Lysipomia brachysiphonia
Lysipomia pumila
Lysipomia sp. nov. 128
Lysipomia glandulifera
Lysipomia sp. nov. 8728
Lysipomia sp. nov. 8743
Lysipomia delicatula
Lysipomia sp. nov. 1163
Lysipomia sp. nov. 8869
Lobelia erinus - HQ823435
8.95
*
1.88
1.26
0.61
1
2.51
1.92
0.59
0.81
1.15
3.4
7.13
5.34
0.91
1.43
3.26
5.03
3.61
7.43
9.54
10.87
9.02
3.58
5.19
2.53
6.57
0.45
1.93
18.38

g

h

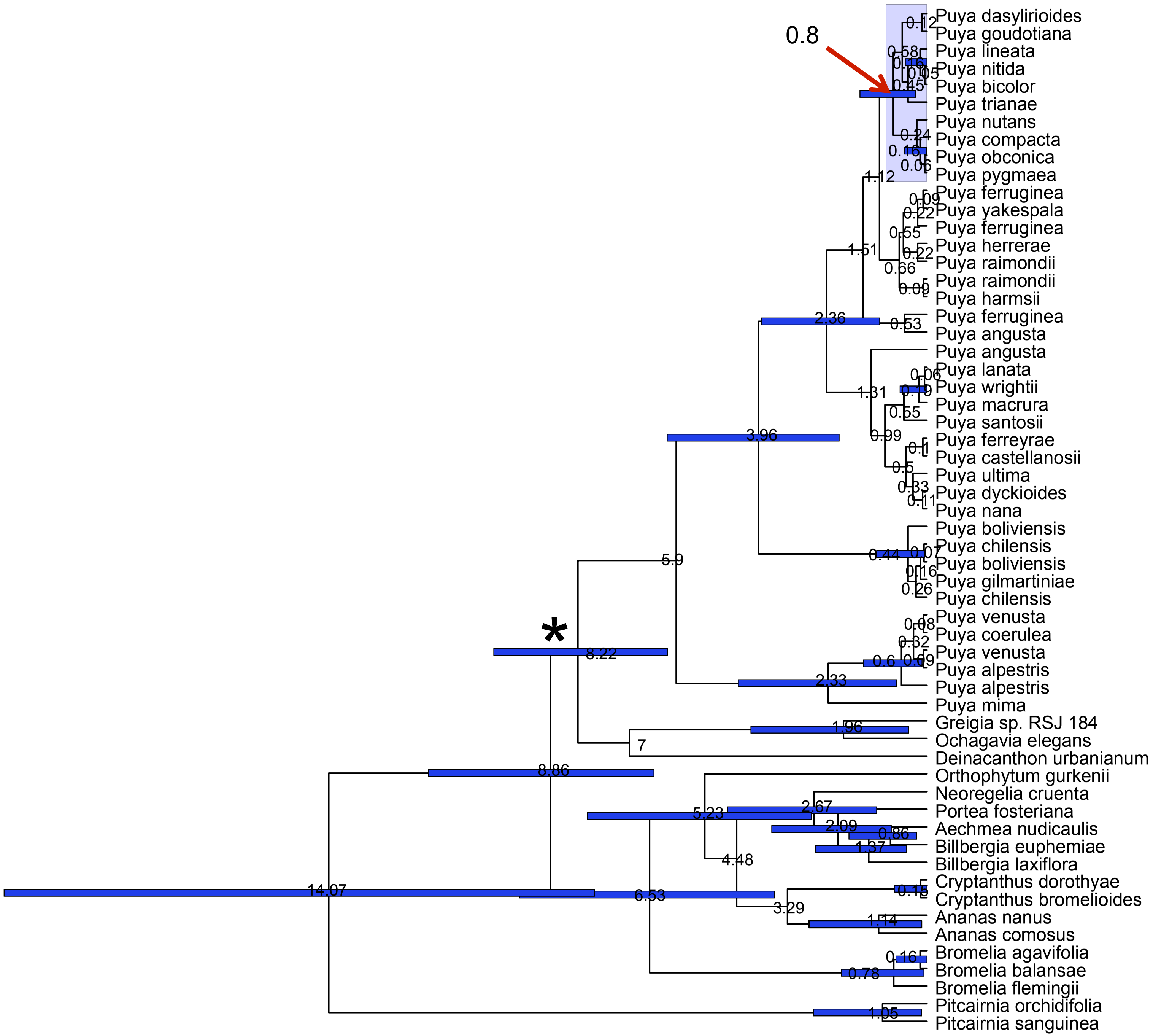
0.8
*
Puya dasylirioides
Puya goudotiana
Puya lineata
Puya nitida
Puya bicolor
Puya trianae
Puya nutans
Puya compacta
Puya obconica
Puya pygmaea
Puya ferruginea
Puya yakespala
Puya ferruginea
Puya herrerae
Puya raimondii
Puya raimondii
Puya harmsii
Puya ferruginea
Puya angusta
Puya angusta
Puya lanata
Puya wrightii
Puya macrura
Puya santosii
Puya ferreyrae
Puya castellanosii
Puya ultima
Puya dyckioides
Puya nana
Puya boliviensis
Puya chilensis
Puya boliviensis
Puya gilmartiniae
Puya chilensis
Puya venusta
Puya coerulea
Puya venusta
Puya alpestris
Puya alpestris
Puya mima
Greigia sp. RSJ 184
Ochagavia elegans
Deinacanthon urbanianum
Orthophytum gurkenii
Neoregelia cruenta
Portea fosteriana
Aechmea nudicaulis
Billbergia euphemiae
Billbergia laxiflora
Cryptanthus dorothyae
Cryptanthus bromelioides
Ananas nanus
Ananas comosus
Bromelia agavifolia
Bromelia balansae
Bromelia flemingii
Pitcairnia orchidifolia
Pitcairnia sanguinea
14.07
8.86
8.22
7
5.9
3.96
2.36
2.33
1.96
1.51
1.31
1.12
0.99
0.66
0.6
0.58
0.55
0.55
0.53
0.5
0.45
0.44
0.33
0.32
0.26
0.24
0.22
0.22
0.16
0.12
0.09
0.08
0.06
0.06
0.05
6.53
5.23
4.48
3.29
2.67
2.09
1.37
1.14
1.05
0.86
0.78
0.16
0.15

Table S1. Details of information for each lineage used in this study.

Lineage	Family	Region	No. of Species	No. of species sampled	Calibration Type	Method of Analysis	Minimum Age (Ma)	Average Age (Ma)	Maximum Age (Ma)	Magallon 0 Min	Magallon 0 Mean	Magallon 0 Max	Magallon 0.9 Min	Magallon 0.9 Mean	Magallon 0.9 Max	Primary Reference	Secondary Reference (s)
Calochortus	Liliaceae	California Floristic Province	38	38	Average ITS rate	Molecular clock	n.a.	7.3	n.a.	n.a.	0.40	n.a.	n.a.	0.20	n.a.	1	1
Calycadenia	Asteraceae	California Floristic Province	11	11	Average ITS rate	Molecular clock (with standard deviation)	4.1	7.64	18.9	0.42	0.22	0.09	0.16	0.08	0.03	1	1
Ceanothus	Rhamnaceae	California Floristic Province	44	2	Rate for *Phylica* (Rhamnaceae)	Molecular clock	n.a.	13.9	n.a.	n.a.	0.22	n.a.	n.a.	0.12	n.a.	1	1
Gilia	Polemoniaceae	California Floristic Province	39	39	Average ITS rate	Molecular clock (with standard deviation)	6.5	12.11	29.9	0.46	0.25	0.10	0.23	0.13	0.05	1	1
Linanthus-Leptosiphon clade	Polemoniaceae	California Floristic Province	23	23	Average ITS rate	Molecular clock (with standard deviation)	9.5	17.58	43.4	0.26	0.14	0.06	0.12	0.06	0.03	1	1
Lithophragma	Saxifragaceae	California Floristic Province	10	9	Island age	Molecular clock	n.a.	1.47	n.a.	n.a.	1.09	n.a.	n.a.	0.40	n.a.	1	1
Crotalarieae & Podalyrieae	Fabaceae	Cape Floristic Region	633	149	Primary fossil	NPRS	n.a.	44	n.a.	n.a.	0.13	n.a.	n.a.	0.09	n.a.	2	2
Ehrharta	Poaceae	Cape Floristic Region	15	7	Secondary fossil	BEAST (with HPD)	26.24	36.44	38.08	0.08	0.06	0.05	0.03	0.02	0.02	3	3
Heliophileae s.l.	Brassicaceae	Cape Floristic Region	59	25	Secondary fossil	BEAST (with HPD)	0.93	2.6	3.68	3.64	1.30	0.92	2.01	0.72	0.51	4	3
Moraea	Iridaceae	Cape Floristic Region	127	36	Secondary fossil	BEAST (with HPD)	15.35	24.25	26.58	0.27	0.17	0.16	0.17	0.11	0.10	5	3
Muraltia	Polygalaceae	Cape Floristic Region	55	74	Secondary fossil	BEAST (with HPD)	8.59	10.89	16.41	0.39	0.30	0.20	0.21	0.17	0.11	6	3
Pelargonium	Geraniaceae	Cape Floristic Region	160	49	Secondary fossil	BEAST (with HPD)	5.41	12.23	16.48	0.81	0.36	0.27	0.51	0.23	0.17	7	2
Pentaschistis s.l.	Poaceae	Cape Floristic Region	64	49	Secondary fossil	BEAST (with HPD)	5.3	5.55	9.89	0.65	0.62	0.35	0.37	0.35	0.20	8	3
Phylica	Rhamnaceae	Cape Floristic Region	150	10	Island age	NPRS (with standard deviation)	7.22	7.4	7.58	0.60	0.58	0.57	0.38	0.37	0.36	49	49
Protea	Proteaceae	Cape Floristic Region	70	70	Secondary fossil	BEAST (with HPD)	11.2	17.7	27.2	0.32	0.20	0.13	0.18	0.11	0.07	10	10
Restionaceae (African)	Restionaceae	Cape Floristic Region	350	20	Secondary fossil	BEAST (with HPD)	20.61	43.83	49.22	0.25	0.12	0.10	0.17	0.08	0.07	11	11
Satyrium	Orchidaceae	Cape Floristic Region	63	25	Secondary fossil	BEAST (with HPD)	n.a.	14.38	n.a.	n.a.	0.24	n.a.	n.a.	0.13	n.a.	12	3
Tribolium-Karoochloa-Schismus	Poaceae	Cape Floristic Region	24	24	Secondary fossil	BEAST (with HPD)	2.21	4.33	6.02	1.12	0.57	0.41	0.52	0.26	0.19	13	3
Zaluzianskya	Scrophulariaceae	Cape Floristic Region	9	3	Secondary fossil	BEAST (with HPD)	1.95	3.03	4.34	0.77	0.50	0.35	0.27	0.18	0.12	14	3
Andira 1	Fabaceae	Cerrado	3	3	Secondary fossil	BEAST (with HPD)	0.5	1.8	3.4	0.81	0.23	0.12	0.24	0.07	0.03	15	15
Lupinus	Fabaceae	Cerrado	11	5	Secondary fossil	BEAST (with HPD)	0.9	1.9	3.1	1.89	0.90	0.55	0.71	0.34	0.21	15	15
Microliceae	Melastomataceae	Cerrado	180	25	Primary fossil	BEAST (with HPD)	6.2	9.8	14	0.73	0.46	0.32	0.47	0.29	0.21	15	15
Mimosa 2	Fabaceae	Cerrado	3	3	Secondary fossil	BEAST (with HPD)	0.3	1.6	3.4	1.35	0.25	0.12	0.39	0.07	0.03	15	15

Table S1. Continued.

Lineage	Family	Region	No. of Species	No. of species sampled	Calibration Type	Method of Analysis	Minimum Age (Ma)	Average Age (Ma)	Maximum Age (Ma)	Magallon 0 Min	Magallon 0 Mean	Magallon 0 Max	Magallon 0.9 Min	Magallon 0.9 Mean	Magallon 0.9 Max	Primary Reference	Secondary Reference(s)
Mimosa 3	Fabaceae	Cerrado	34	16	Secondary fossil	BEAST (with HPD)	2.2	4.4	6.7	1.29	0.64	0.42	0.64	0.32	0.21	15	15
Mimosa 4	Fabaceae	Cerrado	11	6	Secondary fossil	BEAST (with HPD)	0.6	1.6	2.8	2.84	1.07	0.61	1.07	0.40	0.23	15	15
Mimosa 6	Fabaceae	Cerrado	4	2	Secondary fossil	BEAST (with HPD)	0.00001	0.9	2.3	69314.72	0.77	0.30	20256.22	0.23	0.09	15	15
Mimosa 7	Fabaceae	Cerrado	8	6	Secondary fossil	BEAST (with HPD)	1.4	3.2	5.3	0.99	0.43	0.26	0.34	0.15	0.09	15	15
Mimosa 8	Fabaceae	Cerrado	50	26	Secondary fossil	BEAST (with HPD)	2	4.1	6.5	1.61	0.79	0.50	0.86	0.42	0.27	15	15
Mimosa 9	Fabaceae	Cerrado	27	8	Secondary fossil	BEAST (with HPD)	4.3	8.4	12.8	0.61	0.31	0.20	0.29	0.15	0.10	15	15
Tetramolopium	Asteraceae	Hawaiian Archipelago	11	11	not available	Isozyme electrophoretic data	0.6	0.65	0.7	2.84	2.62	2.44	1.07	0.98	0.91	16	17
Geranium	Geraniaceae	Hawaiian Archipelago	5	5	not available	not available	n.a.	2	n.a.	n.a.	0.46	n.a.	n.a.	0.14	n.a.	18	17
Hawaiian Mints	Lamiaceae	Hawaiian Archipelago	57	22	Geology (Bering Straits)	Molecular clock (with standard deviation)	2.6	5	7.4	1.29	0.67	0.45	0.71	0.37	0.25	19	17
Hesperomannia	Asteraceae	Hawaiian Archipelago	3	2	Sang et al. *Dendroseris* rate	Molecular clock	n.a.	4.91	n.a.	n.a.	0.08	n.a.	n.a.	0.02	n.a.	20	17
*Kokia**	Malvaceae	Hawaiian Archipelago	4	1	Average chloroplast rate	Average branch length to crown node/average rate	n.a.	3	n.a.	n.a.	0.23	n.a.	n.a.	0.07	n.a.	21	17
Lobelioideae	Campanulaceae	Hawaiian Archipelago	126	23	Secondary fossil	Penalized likelihood (with standard deviation)	10.49	13.6	16.71	0.39	0.30	0.25	0.24	0.19	0.15	22	17
Metrosideros	Myrtaceae	Hawaiian Archipelago	5	5	Island age	Molecular clock (with standard deviation)	0.5	0.75	1	1.83	1.22	0.92	0.56	0.37	0.28	23	17
Silversword alliance	Asteraceae	Hawaiian Archipelago	30	20	Mediterranean climate	Molecular clock (with standard deviation)	4.4	5.2	6	0.62	0.52	0.45	0.30	0.25	0.22	24	17
Viola	Violaceae	Hawaiian Archipelago	10	8	Island age	island age gives upper age limit to diversification	n.a.	3.7	n.a.	n.a.	0.43	n.a.	n.a.	0.16	n.a.	25	17
Aquilegia	Ranunculaceae	Mediterranean Floristic Province	21	13	Secondary fossil	BEAST (with HPD)	1.25	2.54	3.96	1.88	0.93	0.59	0.84	0.41	0.26	26	26
Cistus-Halimium complex	Cistaceae	Mediterranean Floristic Province	33	28	Primary fossil	Penalized likelihood (with maximum and minimum)	0.14	2.11	4.93	20.02	1.33	0.57	9.88	0.66	0.28	27	28
Dianthus	Caryophyllaceae	Mediterranean Floristic Province	200	104	Primary and secondary fossil	BEAST (with HPD)	0.61	1.095	1.58	7.55	4.21	2.91	4.90	2.73	1.89	28	28
*Erodium***	Geraniaceae	Mediterranean Floristic Province	53	16	Primary fossil	Multidivtime (with HPD)	20.38	24.36	28.34	0.16	0.13	0.12	0.09	0.07	0.06	29	29
*Geranium***	Geraniaceae	Mediterranean Floristic Province	54	14	Primary fossil	Multidivtime (with HPD)	14.65	18.41	21.63	0.22	0.18	0.15	0.12	0.10	0.08	29	29
Narcissus	Amaryllidaceae	Mediterranean Floristic Province	80	38	Secondary fossil and geology	BEAST (with HPD)	14.28	17.8	21.32	0.26	0.21	0.17	0.15	0.12	0.10	30	30
Reseda sect. *Glaucoreseda*	Resedaceae	Mediterranean Floristic Province	5	5	Primary fossil	Penalized likelihood (with standard deviation)	0.12	0.6	1.08	7.64	1.53	0.85	2.32	0.46	0.26	31	31
Ruta	Rutaceae	Mediterranean Floristic Province	9	9	Primary fossil	BEAST (with HPD)	10.37	20	30.88	0.15	0.08	0.05	0.05	0.03	0.02	32	32

Table S1. Continued.

Lineage	Family	Region	No. of Species	No. of species sampled	Calibration Type	Method of Analysis	Minimum Age (Ma)	Average Age (Ma)	Maximum Age (Ma)	Magallon 0 Min	Magallon 0 Mean	Magallon 0 Max	Magallon 0.9 Min	Magallon 0.9 Mean	Magallon 0.9 Max	Primary Reference	Secondary Reference(s)
Aragoa	**Plantaginaceae**	**Páramo**	**17**	**5**	**Island age**	**BEAST (with HPD)**	**0.12**	**0.42**	**0.92**	**17.83**	**5.10**	**2.33**	**7.52**	**2.15**	**0.98**	**33**	**34**
Arcytophyllum	**Rubiaceae**	**Páramo**	**14**	**12**	**Secondary fossil**	**BEAST (with HPD)**	**6.48**	**10.96**	**16.36**	**0.30**	**0.18**	**0.12**	**0.12**	**0.07**	**0.05**	**35**	**35**
Berberis	**Berberidaceae**	**Páramo**	**32**	**10**	**Secondary fossil**	**BEAST (with HPD)**	**0.07**	**3.8**	**9.7**	**39.61**	**0.73**	**0.29**	**19.41**	**0.36**	**0.14**	**36**	**36**
Calceolaria	**Calceolariaceae**	**Páramo**	**65**	**23**	**Secondary fossil**	**BEAST (with HPD)**	**1.42**	**2.45**	**3.51**	**2.45**	**1.39**	**0.99**	**1.37**	**0.78**	**0.56**	**37**	**37**
Draba	**Brassicaceae**	**Páramo**	**55**	**24**	**Secondary fossil**	**BEAST (with HPD)**	**1.6**	**3.05**	**4.1**	**2.07**	**1.09**	**0.81**	**1.13**	**0.59**	**0.44**	**38**	*****
**Espeletiinae**	**Asteraceae**	**Páramo**	**120**	**22**	**Primary fossil**	**BEAST (with HPD)**	**2.42**	**4.04**	**5.92**	**1.69**	**1.01**	**0.69**	**1.04**	**0.62**	**0.42**	**39**	**39**
Festuca	**Poaceae**	**Páramo**	**36**	**5**	**Secondary fossil**	**BEAST (with HPD)**	**1.87**	**4.28**	**7.66**	**1.55**	**0.68**	**0.38**	**0.78**	**0.34**	**0.19**	*****	**40**
Gaultheria	Ericaceae	Páramo	19	8	Average mutation rate	BEAST (with HPD)	2.32	4.59	7.78	0.97	0.49	0.29	0.42	0.21	0.13	*	*
Gentianella	Gentianaceae	Páramo	48	16	Average ITS rate	BEAST (with HPD)	1.6	2.3	3	1.99	1.38	1.06	1.06	0.73	0.56	41	28
Halenia	Gentianaceae	Páramo	43	12	Average ITS rate	BEAST (with HPD)	0.39	0.65	1.28	7.87	4.72	2.40	4.09	2.46	1.25	*	*
Jamesonia+Eriosorus	**Pteridaceae**	**Páramo**	**32**	**2**	**Secondary fossil**	**Penalized likelihood**	**n.a.**	**7.6**	**n.a.**	**n.a.**	**0.36**	**n.a.**	**n.a.**	**0.18**	**n.a.**	**42**	**43**
Lachemilla	Rosaceae	Páramo	35	13	Average ITS rate	BEAST (with HPD)	2.59	3.66	4.93	1.11	0.78	0.58	0.55	0.39	0.29	*	*
Lupinus	**Fabaceae**	**Páramo**	**66**	**6**	**Secondary fossil**	**Penalized likelihood (with standard deviation)**	**1.18**	**1.47**	**1.76**	**2.96**	**2.38**	**1.99**	**1.66**	**1.34**	**1.12**	**44**	**28**
Lysipomia	**Campanulaceae**	**Páramo**	**27**	**20**	**Secondary fossil**	**BEAST (with HPD)**	**6.61**	**8.95**	**11.19**	**0.39**	**0.29**	**0.23**	**0.19**	**0.14**	**0.11**	**45**	**45**
Oreobolus	**Cyperaceae**	**Páramo**	**5**	**5**	**Island age**	**BEAST (with HPD)**	**1.67**	**3.01**	**4.71**	**0.55**	**0.30**	**0.19**	**0.17**	**0.09**	**0.06**	**46**	**44**
Puya	**Bromeliaceae**	**Páramo**	**46**	**10**	**Secondary fossil**	**BEAST (with HPD)**	**0.26**	**0.8**	**1.58**	**12.06**	**3.92**	**1.98**	**6.36**	**2.07**	**1.05**	**47**	*****
Senecio	Asteraceae	Páramo	68	4	Average ITS rate	BEAST (with HPD)	1.21	1.52	1.83	2.91	2.32	1.93	1.64	1.31	1.09	*	*
Valeriana	**Valerianaceae**	**Páramo**	**53**	**20**	**Secondary fossil**	**Multidivtime (with HPD)**	**9.46**	**14.58**	**19.69**	**0.35**	**0.22**	**0.17**	**0.19**	**0.12**	**0.09**	**48**	**48**
Allocasuarina	Casuarinaceae	Southwest Australia	58	34	Primary fossil	Penalized likelihood (with SD)	24	26	36	0.14	0.13	0.09	0.08	0.07	0.05	49	49
Banksia (inc. *Dryandra*)	Proteaceae	Southwest Australia	160	81	Primary fossil	BEAST (with HPD)	36.6	44.5	51.9	0.12	0.10	0.08	0.08	0.06	0.05	50	50
Bossiaea	Fabaceae	Southwest Australia	50	11	Secondary fossil	Penalized likelihood (with SD)	7.22	16.91	31.21	0.45	0.19	0.10	0.24	0.10	0.06	49	51
Daviesia	Fabaceae	Southwest Australia	120	25	Secondary fossil	Penalized likelihood (with SD)	13.75	27.18	43.11	0.30	0.15	0.09	0.18	0.09	0.06	49	51
Pultenea (sensu lato)	Fabaceae	Southwest Australia	100	11	Secondary fossil	Penalized likelihood (with SD)	20.65	35.98	49.98	0.19	0.11	0.08	0.11	0.06	0.05	49	51

Table S1. Continued.

Lineage	Family	Region	No. of Species	No. of species sampled	Calibration Type	Method of Analysis	Minimum Age (Ma)	Average Age (Ma)	Maximum Age (Ma)	Magallon 0 Min	Magallon 0 Mean	Magallon 0 Max	Magallon 0.9 Min	Magallon 0.9 Mean	Magallon 0.9 Max	Primary Reference	Secondary Reference (s)
"Core" Ruschioideae	Aizoaceae	Succulent Karoo	1563	91	Secondary fossil	BEAST (with HPD)	0.6	3.8	7	11.10	1.75	0.95	8.34	1.32	0.72	52	28
Ehrharta	Poaceae	Succulent Karoo	13	13	Secondary fossil	BEAST (with HPD)	5.16	10	11.27	0.36	0.19	0.17	0.14	0.07	0.07	53	3
Heliophileae s.l.	Brassicaceae	Succulent Karoo	18	18	Secondary fossil	BEAST (with HPD)	0.58	1.49	2.09	3.79	1.47	1.05	1.62	0.63	0.45	4	3
Melianthus	Melianthaceae	Succulent Karoo	3	3	Secondary fossil	BEAST (with HPD)	1.46	9.32	9.5	0.28	0.04	0.04	0.08	0.01	0.01	54	3
Moraea	Iridaceae	Succulent Karoo	65	22	Secondary fossil	BEAST (with HPD)	11.27	15.11	20.39	0.31	0.23	0.17	0.17	0.13	0.10	5	3
Muraltia	Polygalaceae	Succulent Karoo	45	2	Secondary fossil	BEAST (with HPD)	1.3	2.51	3.97	2.40	1.24	0.78	1.26	0.65	0.41	6	3
Pelargonium	Geraniaceae	Succulent Karoo	50	36	Secondary fossil	BEAST (with HPD)	n.a.	17.36	n.a.	n.a.	0.19	n.a.	n.a.	0.10	na	7	2
Pentaschistis s.l.	Poaceae	Succulent Karoo	9	8	Secondary fossil	BEAST (with HPD)	0.89	1.56	3.59	1.69	0.96	0.42	0.60	0.34	0.15	8	3
Zaluzianskya	Scrophulariaceae	Succulent Karoo	19	9	Secondary fossil	BEAST (with HPD)	1.95	3.03	4.34	1.15	0.74	0.52	0.50	0.32	0.23	14	3

For each lineage we provide total number of species and number sampled, calibration type, analysis method, crown node ages, diversification rates and references for studies utilized in this analysis. Páramo lineages in bold were those used in the final analysis. * = this work; NPRS = non-parametric rate smoothing; HPD = highest posterior density: SD = standard deviation; n.a. = not available; Magallón 0 (no extinction) = *r*; Magallón 0.9 (extinction)

Table S2. Summary of statistics indicating that the fastest evolving lineages are more likely to be in Páramo than in any other hotspot.

Regions	ProbMean	ProbCI.25	ProbCI.97.5	MaxCountMean	MaxCountCI.25	MaxCountCI.97.5
California Floristic Province	0.000007	0	0	1	1	1
Cape Floristic Region	0.000048	0	0.001	1	1	1
Cerrado	0	0	0	N.A.	N.A.	N.A.
Hawaiian Archipelago	0.075160	0.060	0.092	1.000132088	1	1
Mediterranean Floristic Province	0.395710	0.364	0.427	1.044911323	1.025184194	1.068462114
Páramo	0.508597	0.476	0.539	1.172352947	1.139311777	1.207764513
Southwest Australia	0	0	0	N.A.	N.A.	N.A.
Succulent Karoo	0.020478	0.012	0.03	1.135663207	1	1.307692308

N.A. = Not Applicable

SUPPLEMENTARY REFERENCES

1. Calsbeek, R., Thompson, J. N. and Richardson, J. E. (2003). Patterns of molecular evolution and diversification in a biodiversity hotspot: the California Floristic Province. *Mol. Ecol.* 12, 1021–1029.
2. Edwards, D. and Hawkins, J. A. (2007). Are Cape floral clades the same age? Contemporaneous origins of two lineages in the genistoids s.l. (Fabaceae). *Mol. Phylogenet. Evol.* 45, 952–970.
3. Verboom, G. A, Archibald, J. K., Bakker, F. T., Bellstedt, D. U., Conrad, F., Dreyer, L. L., Forest, F., Galley, C., Goldblatt, P., Henning, J. F., Mummenhoff, K., Linder, H. P., Muasya, A. M., Oberlander, K. C., Savolainen, V., Snijman, D. A., van der Niet T. and Nowell T. L. (2009). Origin and diversification of the Greater Cape flora: Ancient species repository, hot-bed of recent radiation, or both? *Mol. Phylogenet. Evol.* 51, 44–53.
4. Mummenhoff, K., Al-Shehbaz, I., Linder, H. P., Bakker, F. T., Mühlhausen, A. (2005). Phylogeny, morphological evolution, and speciation of endemic Brassicaceae genera in the Cape flora, southern Africa. *Ann. Mo. Bot. Gard.* 92, 400–424.
5. Goldblatt, P., Savolainen, V., Porteous, O. Sostaric, I., Powell, M. Reeves, G., Manning, J. C., Barraclough, T. G. & Chase, M. W. (2002). Radiation in the Cape flora and the phylogeny of peacock irises *Moraea* (Iridaceae) based on four plastid DNA regions. *Mol. Phylogenet. Evol.* 25, 341–360.
6. Forest, F., Nänni, I., Chase, M. W., Crane, P. R. and Hawkins, J. A. (2007). Diversification of a large genus in a continental biodiversity hotspot: temporal and spatial origin of *Muraltia* (Polygalaceae) in the Cape of South Africa. *Mol. Phylogenet. Evol.* 43, 60–74.
7. Bakker, F. T., Culham, A., Marais, E. M. and Gibby, M. (2005) in *Plant Species-level Systematics: new perspectives on pattern & process*, eds. F. T. Bakker, L. W. Chatrou, B. Gravendeel, P. B. Pelser (Ruggell, Gantner Verlag), 75–100.
8. Galley, C. and Linder, H. P. (2007). The phylogeny of the *Pentaschistis* clade (Danthonioideae, Poaceae) based on chloroplast DNA, and the evolution of complex characters. *Evolution* 61, 864–884.
9. Richardson, J. E., Pennington, R. T., Pennington, T. D. & Hollingsworth, P. M. (2001). Rapid and recent origin of species richness in the Cape flora of South Africa. *Nature* 412, 181–183.
10. Valente, L. M., Savolainen, V. and Vargas, P. (2010). Diversification of the African genus *Protea* (Proteaceae) in the Cape biodiversity hotspot and beyond: equal rates in different biomes. *Evolution* 64, 745–760.
11. Linder, H. P., Hardy, C. R. and Rutschmann, F. (2005). Taxon sampling effects in molecular clock dating: an example from the African Restionaceae. *Mol. Phylogenet. Evol.* 35, 569–582.
12. van der Niet, T. and Linder, H. P. (2008). Dealing with incongruence in the quest for the species tree: a case study from the orchid genus *Satyrium*. *Mol. Phylogenet. Evol.* 47, 154–174.

13. Verboom, G. A. (2006). A phylogeny of the schoenoid sedges (Cyperaceae: Schoeneae) based on plastid DNA sequences, with special reference to the genera found in Africa. *Mol. Phylogenet. Evol.* 38, 79–89.
14. Archibald, J. K., Mort, M. E. and Wolfe, A. D. (2005). Phylogenetic relationships within *Zaluzianskya* (Scrophulariaceae s.s., tribe Manuleeae): classification based on DNA sequences from multiple genomes and implications for character evolution and biogeography. *Syst. Bot.* 30, 196–215.
15. Simon, M. F., Gretherc, R., de Queiroz, L. P., Skema, C., Pennington, R. T. & Hughes, C. E. (2009). Recent assembly of the Cerrado, a neotropical plant diversity hotspot, by in situ evolution of adaptations to fire. *Proc. Natl. Acad. Sci. USA* 106, 20359–20364.
16. Lowrey, T. K. (1995) in *Hawaiian biogeography: evolution on a hotspot archipelago*, eds. W. L. Wagner and V. A. Funk (Washington, Smithsonian Institution Press), 195–221.
17. Price, J. P. and Wagner, W. L. (2004). Speciation in Hawaiian angiosperm lineages: cause, consequence and mode. *Evolution* 58, 2185–2200.
18. Pax, D. L., Price, R. A. and Michaels, H. J. (1997). Phylogenetic position of the Hawaiian geraniums based on *rbcL* sequences. *Am. J. Bot.* 84, 72–78.
19. Lindqvist, C. and Albert, V. A. (2002). Origin of the endemic Hawaiian mints within North American *Stachys* (Lamiaceae). *Am. J. Bot.* 89, 1709–1724.
20. Kim, H. G., Keeley, S. C., Vroom, P. S. and Jansen, R. K. (1998). Molecular evidence for an African origin of the Hawaiian endemic *Hesperomannia* (Asteraceae). *Proc. Natl. Acad. Sci. USA* 5, 15440–15445.
21. Seelanan, T., Schnabel, A. and Wendel, J. F. (1997). Congruence and consensus in the cotton tribe (Malvaceae). *Syst. Bot.* 22, 259–290.
22. Givnish, T. J. (2009). Origin, adaptive radiation and diversification of the Hawaiian lobeliads (Asterales: Campanulaceae). *Proc. R. Soc. Lond. B. Biol. Sci.* 276, 407–416.
23. Wright, S. D., Yong, C. G., Wichman, S. R., Dawson, J. W. and Gardner, R. C. (2001). Stepping stones to Hawaii: a trans-equatorial dispersal pathway for *Metrosideros* (Myrtaceae) inferred from nrDNA (ITS 1 ETS). *J. Biogeogr.* 28, 769–774.
24. Baldwin, B. G. and Sanderson, M. J. (1998). Age and rate of diversification of the Hawaiian silversword alliance (Compositae). *Proc. Natl. Acad. Sci. USA* 95, 9402–9406.
25. Ballard, H. E. and Sytsma, K. J. (2000). Evolution and biogeography of the woody Hawaiian violets (*Viola*, Violaceae): Arctic origins, herbaceous ancestry and bird dispersal. *Evolution* 54, 1521–1532.
26. Bastida, J. M., Alcátara, J. M., Rey, P. J., Vargas, P. and Herrera, C. M. (2010). Extended phylogeny of *Aquilegia*: the biogeographical and ecological patterns of two simultaneous but contrasting radiations. *Plant Syst. Evol.* 284, 171–185.
27. Guzmán, B., Lledó, M. D. and Vargas, P. (2009). Adaptive radiation in Mediterranean *Cistus* (Cistaceae). *PLoS One* 4, e6362.
28. Valente, L. M., Savolainen, V. and Vargas, P. (2010). Unparalleled rates of species diversification in Europe. *Proc. R. Soc. Lond. B Biol. Sci.* 277, 1489–1496.

29. Fiz-Palacios, O., Vargas, P., Vila, R., Papadopulos, A. S. T., Aldasoro, J. J. (2011).The uneven phylogeny and biogeography of *Erodium* (Geraniaceae): radiations in the Mediterranean and recent recurrent intercontinental colonization. *Ann. Bot.* 106, 871–84.
30. Santos-Gally, R., Vargas, P. and Arroyo, J. (2011). Insights into Neogene Mediterranean biogeography based on phylogenetic relationships of mountain and lowland lineages of *Narcissus* (Amaryllidaceae). *J. Biogeogr.* 39, 782–798.
31. Martín-Bravo, S., Valcárcel, V., Vargas, P. and Luceño, M. (2010). Geographical speciation related to Pleistocene range shifts in the western Mediterranean mountains (*Reseda* sect. *Glaucoreseda*, Resedaceae). *Taxon* 59, 466–482.
32. Salvo, G., Ho, S. Y. H., Rosenbaum, G., Ree, R. and Conti, E. (2010). Tracing the temporal and spatial origins of island endemics in the Mediterranean region: a case study from the citrus family (*Ruta* L., Rutaceae). *Syst. Biol.* 59, 705–22.
33. Bello, M. A., Chase, M. W., Olmstead, R. G., Rønsted, N. and Albach, D. (2002). The Páramo endemic *Aragoa* is the sister genus of *Plantago* (Plantaginaceae, Lamiales): evidence from plastid *rbc*L and nuclear ribosomal ITS sequence data. *Kew Bull.* 57, 585–597.
34. Rønsted, N., Chase, M. W., Albach, D. C. and Bello, M. A. (2002). Phylogenetic relationships within *Plantago* (Plantaginaceae): evidence from nuclear ribosomal ITS and plastid trnL-F sequence data. *Bot. J. Linn. Soc.* 139, 323–338.
35. Bremer, B. and Erikson, T. (2009). Time Tree of Rubiaceae: Phylogeny and Dating the Family, Subfamilies, and Tribes. *Int. J. Plant Sci.* 170, 766–793.
36. Adhikari, B. (2010). Phylogeography of *Berberis* in Nepal. PhD thesis, University of Edinburgh.
37 Renner, S. S. and Schaefer, H. (2010). The evolution and loss of oil-offering flowers -- new insights from dated phylogenies for plants and bees. *Philos. Trans. R. Soc. Lond. B Biol. Sci.* 365, 423–435.
38. Couvreur, T. L. P., Franzke, A., Al-Shehbaz, I. A., Bakker, F. T., Koch, M. A. and Mummenhoff, K. (2010). Molecular phylogenetics, temporal diversification, and principles of evolution in the mustard family (Brassicaceae). *Mol. Biol. Evol.* 27, 55–71.
39. Rauscher, J. T. (2002). Molecular phylogenetics of the *Espeletia* complex (Asteraceae): evidence from nrDNA ITS sequences on the closest relatives of an Andean adaptive radiation. *Am. J. Bot.* 89, 1074–1084.
49. Inda, L. A., Segarra-Moragues, J. G., Muller, J., Peterson, P. M. and Catalan, P. (2008). Dated historical biogeography of the temperate Loliinae (Poaceae, Pooideae) grasses in the northern and southern hemispheres. *Mol. Phylogenet. Evol.* 46, 932–957.
41. von Hagen, K. B. and Kadereit, J. W. (2001). The phylogeny of *Gentianella* (Gentianaceae) and its rapid colonization of the southern hemisphere as revealed by nuclear and chloroplast DNA sequence variation. *Org. Divers. Evol.* 1, 61–79.
42. Sánchez-Baracaldo, P. (2004). Phylogenetics and biogeography of the tropical fern genera *Jamesonia* and *Eriosorus* (Pteridaceae). *Am. J. Bot.* 91, 274–284.
43. Schuettpelz, E. and Pryer, K. M. (2009). Evidence for a Cenozoic radiation of ferns in an angiosperm-dominated canopy. *Proc. Natl. Acad. Sci. USA* 105, 11200–11205.

44. Pennington, R. T., Lavin, M., Hughes, C., Sarkinen, T., Lewis, G., Klitgaard, B. (2010). Differing diversification histories in the Andean biodiversity hotspot. *Proc. Natl. Acad. Sci. USA* 107, 13783-13787.
45. Antonelli, A. (2009). Have giant lobelias evolved several times independently? Life form shifts and historical biogeography of the cosmopolitan and highly diverse subfamily Lobelioideae (Campanulaceae). *BMC Biol.* 7, 82–103.
46. Chacón, J., Madriñán, S., Chase, M. W. and Bruhl, J. (2006). Molecular phylogenetics of *Oreobolus* (Cyperaceae) and the origin and diversification of the American species. *Taxon* 55, 359-366.
47. Schmidt-Jabaily, R. and Sytsma, K. J. (2010). Phylogenetics of *Puya* (Bromeliaceae): placement, major lineages, and evolution of Chilean species. *Am. J. Bot.* 97, 337–356.
48. Bell, C. D. and Donoghue, M. J. (2005). Phylogeny and biogeography of the Valerianaceae (Dipsacales) with special reference to the South American valerians. *Org. Divers. Evol.* 5, 147–159.
49. Crisp, M. D., Cook, L. G. and Steane, D. A. (2004). Radiation of the Australian flora: what can comparisons of molecular phylogenies across multiple taxa tell us about the evolution of diversity in present-day communities? *Philos. Trans. R. Soc. Lond. B Biol. Sci.* 359, 1551–1571.
50. He, T., Lamont, B. B. and Downes, K. S. (2011). *Banksia* born to burn. *New. Phytol.* 191, 184–96.
51. Wojciechowski, M. F. (2002) in *Advances in legume systematics, part 10: higher level systematics*, eds. B. B. Klitgaard and A. Bruneau (Kew, The Royal Botanic Gardens), 5–35.
52. Klak, C., Reeves, G. and Hedderson, T. (2004). Unmatched tempo of evolution in southern African semi-desert ice plants. *Nature* 427, 63–65.
53. Verboom, G. A., Linder, H. P. and Stock, W. D. (2003). Phylogenetics of the grass genus *Ehrharta* Thunb.: evidence for radiation in the summer-arid zone of the South African Cape. *Evolution* 57, 1008–1021.
54. Linder, H. P., Dlamini, T. S., Henning, J. and Verboom, G. A. (2006). The evolutionary history of *Melianthus* (Melianthaceae). *Am. J. Bot.* 93, 1052–1064.

APPENDIX S1

Genbank accession numbers for taxa sampled (n.a. = not available).

Aragoa: *Aragoa abietina* AJ459404, *Aragoa cundinamarcensis* AJ459403, *Aragoa cupressina* AJ459402, *Aragoa xchingacensis* AJ459401, *Aragoa xfunzana* AJ459400, *Plantago afra* AY101892, *Plantago albicans* AY101905, *Plantago alpina* AY101877, *Plantago amplexicaulis* AY101900, *Plantago arborescens* AY101886, *Plantago arenaria* AY101891, *Plantago aristata* AY101911, *Plantago asiatica* AY101862, *Plantago atrata* AY101895, *Plantago australis* AY101874, *Plantago bellardii* AY101902, *Plantago ciliata* AY101906, *Plantago cornuti* AY101859, *Plantago coronopus* AY101882, *Plantago crassifolia* AY101881, *Plantago cretica* AY101901, *Plantago debilis* AY101868, *Plantago erecta* AY101909, *Plantago famarae* AY101888, *Plantago hookeriana* AY101913, *Plantago lagopus* AY101897, *Plantago lanceolata* AY101898, *Plantago leiopetala* AY101899, *Plantago lundborgii* AY101907, *Plantago macrorhiza* AY101883, *Plantago major* AY101861, *Plantago maritima* AY101879, *Plantago mauritanica* AY101890, *Plantago maxima* AY101864, *Plantago media* AY101865, *Plantago myosuros* AY101873, *Plantago nivalis* AY101896, *Plantago ovata* AY101903, *Plantago palmata* AY101860, *Plantago patagonica* AY101912, *Plantago raoulii* AY101867, *Plantago reniformis* AY101858, *Plantago rigida* AY101876, *Plantago rugelii* AY101863, *Plantago sarcophylla* AY101893, *Plantago sempervirens* AY101889, *Plantago sericea* AY101910, *Plantago serraria* AY101880, *Plantago spathulata* AY101869, *Plantago squarrosa* AY101894, *Plantago stauntonii* AY101870, *Plantago stocksii* AY101904, *Plantago subspathulata* AY101884, *Plantago subulata* AY101878, *Plantago tandilensis* AY101908, *Plantago tenuiflora* AY101866, *Plantago tomentosa* AY101872, *Plantago trinitatis* AY101871, *Plantago uniflora* AY101885, *Plantago uniglumis* AY101875, *Plantago webbii* AY101887, *Veronica chamaedrys* AF313003, *Veronica fruticulosa* AF313004, *Veronica glandulosa* AF313008.

Arcytophyllum: *Arcytophyllum aristatum* AM182061, *Arcytophyllum capitatum* AM182062, *Arcytophyllum capitatum* AM182063, *Arcytophyllum ciliolatum* AM182064, *Arcytophyllum filiforme* AM182065, *Arcytophyllum filiforme* AM182066, *Arcytophyllum lavarum* AM182067, *Arcytophyllum muticum* AM182070, *Arcytophyllum peruvianum* AM182068, *Arcytophyllum peruvianum* AM182069, *Arcytophyllum rivetii* AM182071, *Arcytophyllum setosum* AM182072, *Arcytophyllum setosum* AM182073, *Arcytophyllum thymifolium* AM182074, *Arcytophyllum thymifolium* AM182075, *Arcytophyllum vernicosum* AM182076, *Paederia bojeriana* FJ695454, *Phyllis nobla* AF257939.

Berberis*:* Not submitted.

Calceolaria: *Calceolaria atahualpae* AJ579420, *Calceolaria ballotifolia* AJ579434, *Calceolaria barbata* AJ579409, *Calceolaria bicrenata* AJ579422, *Calceolaria biflora* FJ527784, *Calceolaria boliviana* AJ579404, *Calceolaria brachiata* AJ579462, *Calceolaria brunellifolia* FJ527795, *Calceolaria buchtieniana* AJ579411, *Calceolaria calycina* AJ579444, *Calceolaria cavanillesii* FJ527791, *Calceolaria chelidonioides* AJ579441, *Calceolaria comosa* AJ579427, *Calceolaria*

connatifolia AJ579469, *Calceolaria corymbosa* AJ579393, *Calceolaria corymbosa subsp. floccosa* FJ527790, *Calceolaria crenatiflora* FJ527786, *Calceolaria cypripediiflora* AJ579405, *Calceolaria dentata* FJ527801, *Calceolaria dentifolia* AJ579450, *Calceolaria dichotoma* AJ579437, *Calceolaria dilatata* AJ579464, *Calceolaria elatior* FJ527788, *Calceolaria engleriana* AJ579401, *Calceolaria ericoides* AJ579408, *Calceolaria filicaulis* AJ579386, *Calceolaria filicaulis* AJ579394, *Calceolaria fusca* AJ579466, *Calceolaria gaultherioides* AJ579451, *Calceolaria germainii* AJ579387, *Calceolaria glacialis* FJ527803, *Calceolaria glauca* AJ579423, *Calceolaria gossypina* AJ579459, *Calceolaria helianthemoides* AJ579461, *Calceolaria inflexa* AJ579418, *Calceolaria integrifolia* AJ579396, *Calceolaria jujuyensis* FJ527789, *Calceolaria lagunae* FJ527793, *Calceolaria lamiifolia* AJ579435, *Calceolaria lanata* AJ579447, *Calceolaria lanceolata* FJ527797, *Calceolaria lavandulifolia* AJ579460, *Calceolaria lehmanniana* AJ579454, *Calceolaria linearis* AJ579410, *Calceolaria lobata* AJ579428, *Calceolaria maculata* AJ579433, *Calceolaria mandoniana* AJ579442, *Calceolaria martinezii* AJ579456, *Calceolaria mendocina* FJ527800, *Calceolaria microbefaria* AJ579468, *Calceolaria moyobambae* AJ579449, *Calceolaria myriophylla* AJ579403, *Calceolaria nivalis* AJ579465, *Calceolaria oblonga* AJ579414, *Calceolaria obtusa* AJ579432, *Calceolaria parviflora* FJ527802, *Calceolaria parvifolia* AJ579412, *Calceolaria pavonii* AJ579445, *Calceolaria pedunculata* AJ579453, *Calceolaria penlandii* AJ579436, *Calceolaria pennellii* AJ579389, *Calceolaria perfoliata* AJ579443, *Calceolaria petiolaris* FJ527794, *Calceolaria phaceliifolia* AJ579429, *Calceolaria phaeotricha* AJ579470, *Calceolaria pinifolia* AJ579392, *Calceolaria plectranthifolia* AJ579398, *Calceolaria polifolia* AJ579397, *Calceolaria polyclada* AJ579402, *Calceolaria prichardii* FJ527798, *Calceolaria purpurascens* AJ579463, *Calceolaria revoluta* AJ579407, *Calceolaria rivularis* AJ579440, *Calceolaria rosmarinifolia* AJ579457, *Calceolaria rugulosa* AJ579430, *Calceolaria rupestris* AJ579406, *Calceolaria salicifolia* AJ579452, *Calceolaria santolinoides* FJ527787, *Calceolaria scabra* AJ579421, *Calceolaria semiconnata* AJ579467, *Calceolaria sericea* AJ579426, *Calceolaria sp. 1 AC* FJ527785, *Calceolaria sp. 2 AC* FJ527799, *Calceolaria sparsiflora* AJ579417, *Calceolaria spruceana* AJ579458, *Calceolaria stricta* AJ579455, *Calceolaria virgata* AJ579431, *Calceolaria volckmanni* FJ527792, *Calceolaria vulpina* AJ579416, *Jovellana punctata_O* FJ527783, *Jovellana violacea_O* AJ579385, *Porodittia triandra_O* AJ579424.

Draba: *Aethionema arabicum* AY254539, *Alyssoides utriculata* EF514593, *Alyssopsis mollis* GQ424523, *Ammosperma cinerea* GQ424606, *Anastatica hierochuntica* GQ424524, *Anchonium elichrysifolium* DQ357516, *Anelsonia eurycarpa* DQ452059, *Aphragmus oxycarpus* DQ165337, *Arabidella glaucescens* n.a., *Arabidopsis thaliana* AJ232900, *Arabis alpina* DQ060109, *Arcyosperma primulifolium* GQ424525, *Asta schaffneri* GQ424526, *Athysanus pusillus* EF514629, *Aubrieta deltoidea* AJ232909, *Baimashania pulvinata* DQ523426, *Ballantinia antipoda* n.a., *Barbarea verna* X98631, *Berteroa incana* EF514631, *Biscutella didyma* DQ452058, *Boechera stricta* AF137575, *Boreava orientalis* DQ249859, *Bornmuellera baldaccii* EF514635, *Bornmuellera tymphaea* EF514640, *Brassica juncea* AF128093, *Braya rosea* AY353129, *Brossardia*

papyracea GQ497852, *Cakile maritima* DQ249830, *Calepina irregularis* AY722504, *Camelina microcarpa* AF137574, *Capsella bursa pastoris* AF531561, *Cardamine scutata* DQ268478, *Carinavalva glauca* GQ424527, *Carrichtera annua* DQ249829, *Catenulina hedysaroides* GQ424607, *Caulostramina jaegeri* AF137572, *Ceratocnemum rapistroides (=Caulanthus amplexicaulis)* AF346630, *Chalcanthus renifolius* GQ424528, *Chartoloma platycarpum* GQ424529, *Chaunantus acuminatus* GQ497855, *Cheesmania fastigiata* AF100680, *Chlorocrambe hastatus* GQ497856, *Chorispora tenella* DQ357526, *Cithareloma lehmannii* EF514641, *Clastopus vestitus* GQ424608, *Clausia aprica* DQ357529, *Clypeola aspera* EF514642, *Cochlearia danica* HQ268638, *Conringia persica* GQ424570, *Cordylocarpus muricatus* DQ249827, *Crambe filiformis* AY722434, *Cremolobus chilensis* GQ424530, *Crucihimalaya mollissima* AF137552, *Cryptospora falcata* DQ357531, *Cuphonotus humistratus* not found, *Cusickiella quadricostata* DQ452066, *Descurainia sophia* AY230619, *Diceratella inermis* DQ357533, *Didesmus aegypticus* GQ424531, *Didymophysa fedtschenkoana* EF514648, *Dielsiocharis kotschi* GQ424532, *Dimorphocarpa wislizenii* AF137593, *Diplotaxis erucoides* DQ249826, *Diptychocarpus strictus* DQ357534, *Dithyrea californica* AF137592, *Dontostemon integrifolius* DQ357536, *Draba acaulis A Uni* DQ467276, *Draba altaica* AY134115, *Draba alyssoides* DQ467269, *Draba aretioides* DQ467582, *Draba boyacana* AF146500, *Draba boyacana1* GU202451, *Draba bruniifolia A Uni* AY047664, *Draba bruniifolia A1 Uni* DQ467286, *Draba cappadocica A Uni* DQ467590, *Draba cuzcoensis* GU202465, *Draba cuzcoensis1* GU202466, *Draba depressa* AF146458, *Draba depressa1* GU202468, *Draba ellipsoidea A Uni* DQ467338, *Draba ellipsoidea A1 Uni* AY134148, *Draba ellipsoidea A2 Uni* DQ467397, *Draba eriopoda A Uni* DQ467398, *Draba eriopoda A1 Uni* AY134094, *Draba hallii* AF146464, *Draba hallii1* GU202482, *Draba hispida A Uni* DQ467293, *Draba hispida A1 Uni* DQ467296, *Draba hookeri* GU202483, *Draba hookeri1* DQ467585, *Draba hueti Ai Uni* DQ467299, *Draba huetii A Uni* DQ467298, *Draba incompta A Uni* DQ467581, *Draba jucunda A Uni* DQ467346, *Draba longisiliqua A Uni* DQ467240, *Draba matthioloides* AF146503, *Draba matthioloides subsp. saundersii* GU202503, *Draba nemorosa A Uni* JF976276, *Draba nemorosa A1 Uni* JF976275, *Draba nuda A Uni* AF137577, *Draba obovata* DQ467586, *Draba obovata1* GU202516, *Draba oreades A Uni* AY134151, *Draba rosularis A Uni* DQ467259, *Draba rosularis A1 Uni* DQ467258, *Draba schusteri* GU202536, *Draba schusteri1* AF146453, *Draba sibirica A Uni* DQ467462, *Draba sibirica A1 Uni* DQ467463, *Draba splendens* GU202539, *Draba splendens1* AF146475, *Draba violacea* GU202549, *Draba violacea1* AF146507, *Draba wurdackii* AF146477, *Draba wurdackii1* GU202550, *Drabastrum alpestre* n.a., *Dryopetalon runcinatum* AF531634, *Elburzia fenestrata* GQ424533, *Eremobium aegyptiacum* DQ357537, *Eremodraba intricatissima* GQ424534, *Eremophyton chevallieri* GQ424535, *Eruca sativa* DQ249821, *Erucaria hispanica* AY722495, *Erysimum canum* DQ357539, *Euclidium syriacum* DQ357543, *Eutrema heterophyllum* DQ165352, *Farsetia aegyptiaca* EF514649, *Fezia pterocarpa* GQ424536, *Fibigia suffruticosa* FM164657, *Fortuynia garcini* AF263398, *Fourraea alpina* DQ518395, *Galitzkya macrocarpa* EF514655, *Geococcus pusillus* GQ424571, *Glaucocarpum suffrutescens* GQ424567,

Goldbachia laevigata DQ357545, *Gorodkovia jakutica* AY230646, *Graellsia saxifragifolia* GQ424572, *Grammosperma dusenii* GQ424568, *Guiraoa arvensis* AY722468, *Halimolobos diffusus* AF307645, *Harmsiodoxa blennoiodes* n.a., *Hedinia tibetica* AY230607, *Hemicrambe fruticulosa* AY722469, *Henophyton deserti* GQ424537, *Hesperis sibirica* DQ357548, *Hormathophylla longicaulis* EF514660, *Hornungia petraea* AJ440303, *Horwoodia dicksoniae* GQ424538, *Ianhedgea minutiflora* AF137568, *Iberis amara* AJ440311, *Iodanthus pinnatifidus* GQ424539, *Irenepharsus trypherus* n.a., *Isatis tinctoria* DQ249851, *Ischnocarpus exilis* EF015673, *Iti lacustris* AF100683, *Kernera saxatilis* AJ440313, *Lachnocapsa spathulata* GQ424540, *Leavenworthia crassa* GQ424541, *Leiospora eriocalyx* DQ357554, *Lepidium campestris* AF055197, *Lepidostemon glaricola* GQ424542, *Leptaleum filifolium* DQ357556, *Lignariella serpens* DQ165360, *Lobularia maritima* EF514681, *Lunaria rediviva* GQ424543, *Lyrocarpa coulteri* AF137591, *Malcolmia littorea* DQ357559, *Mancoa foliosa* AF307632, *Maresia nana* DQ357562, *Mathewsia foliosa* DQ357563, *Matthiola fragrans* DQ249848, *matthiola sp* GQ424566, *Megadenia pygmaea* GQ424544, *Menkea crassa* n.a., *Microlepidium pilosulum* GQ497869, *Moricandia arvensis* EF601899, *Moriera spinosa* GQ424545, *Morisia monanthos* AY722476, *Mostacillastrum orbignyanum* AF531583, *Murbeckiella huettii* GQ424546, *Myagrum perfoliatum* GQ424547, *Nasturtiopsis coronopifolia* GQ424548, *Nasturtium officinale* X98643, *Neotorularia korolkovii* AY353155, *Nerisyrenia linearifolia* AF137587, *Neslia paniculata* AF137576, *Nevada holmgrenii* DQ452061, *Noccaea fendleri* AY154806, *Notothlaspi rosulatum* AF100690, *Ochthodium aegyptiacum* GQ497870, *Octoceras lehmannianum* GQ424609, *Olimarabidopsis pumila* AF137549, *Oreoloma violaceum* DQ357576, *Oreophyton falcatum* GQ424549, *Ornithocarpa torulosa* GQ424550, *Orychophragmus violaceus* EU306541, *Pachycladon novae zelandiae* EF015677, *Pachyneurum grandiflorum* DQ467584, *Pachypterygium brevipes* GQ131326, *Parlatoria rostrata* GQ424552, *Parolinia intermedia* DQ357577, *Parrya exscapa* FN821614, *Paysonia densipila* AF137586, *Pegaeophyton scapifolium* DQ518398, *Peltaria alliaceae* DQ249855, *Peltariopsis planisiliqua* GQ424553, *Pennellia micrantha* AF307629, *Phaeonychium jafrii* DQ523422, *Phlegmatospermum eremaeum* n.a., *Phoenicaulis cheiranthoides* DQ399121, *Physaria acutifolia* AF137582, *Physoptychis caspica* EF514682, *Planodes virginicum* GQ424554, *Polyctenium fremontii* AY230647, *Pseudoarabidopsis toxophylla* AF137558, *Pseudocamelina glaucophylla* DQ357581, *Psychine stylosa* DQ249835, *Raffenaldia primuloides* AY722478, *Raphanus raphanistrum* AY722479, *Rapistrum rugosum* DQ249825, *Rhammatophyllum afghanicum* DQ357583, *Ricotia cretica* GQ497880, *Romanschulzia spec* AF531636, *Rorippa indica* AF128108, *Rytidocarpus moricandioides* AY722483, *Sameraria nummularia* GQ424555, *Savignya parviflora* AF263399, *Scambopus curvipes* n.a., *Schimpera arabica* GQ424556, *Schivereckia podolica* AY134136, *Schoenocrambe linearifolia* AF531612, *Schouwia purpurea* AY722500, *Selenia dissecta* GQ424557, *Shangrilaia nana* GQ424558, *Sibara rosulata* AF531648, *Sibaropsis hammittii* GQ424559, *Sinapidendron frutescens* DQ249823, *Sinosophiopsis bartholomewii* AY230609, *Sisymbrella aspera* GQ424560, *Sisymbriopsis mollipila* AY353157, *Sisymbrium*

loeselii AF531573, *Smelowskia calycina* AY230640, *Sobolewskia caucasica* GQ424561, *Solms laubachia flabellata* GQ424562, *Solms laubachia zhongdianensis* DQ523415, *Sophiopsis flavissima* AY230611, *Sphaerocardamum macropetalum* AF137589, *Spryginia winkleri* GQ424563, *Stanleya pinnata* AF531620, *Stenopetalum decipiens* GQ424564, *Sterigmostemum acanthocarpum* DQ357591, *Streptanthella longirostris* AF531621, *Streptoloma desertorum* FM164618, *Strigosella africana* DQ357557, *Subularia monticola* GQ424565, *Succowia balearica* AF263395, *Synthlipsis greggii* AF137590, *Taphrospermum altaicum* DQ165364, *Tauscheria lasiocarpa* DQ249843, *Tetracme quadricornis* DQ357602, *Thellungiella salsuginea* AF531626, *Thelypodiopsis ambigua* AF531625, *Thysanocarpus curvipes* AY254542, *Trachystoma balii* AY722492, *Turritis glabra* AJ232922, *Vella pseudocytisus* AF263393, *Warea sessilifolia* AF531644, *Zilla spinosa* AY722501, *Zuvanda crenulata* DQ357606.

Espeletiinae: *Carramboa littlei* AF465868, *Carramboa trujillensis* AF465866, *Coespeletia moritziana* AF465851, *Coespeletia spicata* n.a., *Espeletia anglicana* n.a., *Espeletia boyacensis* n.a., *Espeletia grandiflora* n.a., *Espeletia killipii* n.a., *Espeletia pycnophylla* AF465865, *Espeletia schultzii* AF465864, *Espeletiopsis angustifolia* n.a., *Espeletiopsis caldassi* n.a., *Espeletiopsis jimenez-quesadae* AF465863, *Espeletiopsis pannosa* AF465862, *Espeletiopsis santanderensis* n.a., *Ichthyothere agrestis* AF465861, *Ichthyothere granvillei* AF465860, *Ichthyothere latifolia* AF465857, *Ichthyothere macdanielii* AF465856, *Ichthyothere rufa* AF465855, *Ichthyothere scandens* AF465854, *Ichthyothere terminalis* AF465849, *Libanothamnus humbertii* AF465848, *Paramiflos glandulosa* AF465904, *Ruilopezia atropurpurea* AF465903, *Ruilopezia marcescens* AF465902, *Ruilopezia ruizii* AF465901, *Rumfordia floribunda* AF465900, *Rumfordia guatemalensis* AF465898, *Rumfordia guatemalensis* AF465899, *Rumfordia penninervis* AF465897, *Rumfordia reveali*i AF465896, *Smallanthus connatus* AF465895, *Smallanthus fruticosus* AF465894, *Smallanthus jelskii* AF465893, *Smallanthus maculatus* AF465892, *Smallanthus meridensis* AF465891, *Smallanthus microcephalus* AF465886, *Smallanthus oaxacanus* AF465885, *Smallanthus pyramidalis* AF465884, *Smallanthus quichensis* AF465883, *Smallanthus riparius* AF465882, *Smallanthus siegesbeckius* AF465881, *Smallanthus sonchifolius* AF465880, *Smallanthus uvedalius* AF465879, *Tamania chardonii* AF465875.

Festuca: *Cutandia maritima* EF584915, *Festuca andicola* EF584922, *Festuca chimborazensis* EF584930, *Festuca cochabambana* EF584931, *Festuca cuzcoensis* EF584932, *Festuca flacca* EF584938, *Festuca glumosa* EF584940, *Festuca longivaginata* EF584951, *Festuca nardifolia* EF584954, *Festuca orthophylla* EF584957, *Festuca parodiana* EF584959, *Festuca purpurascens* EF584964, *Festuca rigescens* EF584966, *Festuca subantarctica* EF584973, *Festuca tolucensis* EF584976, *Festuca vaginalis* EF584977.

Lysipomia: *Lysipomia aretioides* AF054964, *Lysipomia bilineata* AF054963, *Lysipomia brachysiphonia* AF054962, *Lysipomia caespitosa* AF054961, *Lysipomia crassomarginata* AF054960, *Lysipomia cuspidata* AF054959, *Lysipomia cylindrocarpa* AF054958, *Lysipomia delicatula* AF054957, *Lysipomia glandulifera* AF054956, *Lysipomia globularis* AF054955, *Lysipomia hutchisonii* AF054954, *Lysipomia laciniata* AF054953, *Lysipomia laricina* AF054952, *Lysipomia*

lehmannii AF054951, *Lysipomia montioides* AF054950, *Lysipomia multiflora* AF054949, *Lysipomia muscoides* AF054948, *Lysipomia oellgaardii* AF054947, *Lysipomia pumila* AF054946, *Lysipomia sp. nov. 1163* AF054966, *Lysipomia sp. nov. 128* AF054940, *Lysipomia sp. nov. 1419* AF054968, *Lysipomia sp. nov. 8728* AF054965, *Lysipomia sp. nov. 8743* AF054967, *Lysipomia sp. nov. 8869* AF054969, *Lysipomia sparrei* AF054945, *Lysipomia speciosa* AF054944, *Lysipomia sphagnophila* AF054943, *Lysipomia subpeltata* AF054942, *Lysipomia vitreola* AF054939.

Puya: *Aechmea nudicaulis* FJ968256, *Ananas comosus* FJ968247, *Ananas nanus* FJ968242, *Billbergia euphemiae* FJ968246, *Billbergia laxiflora* FJ968236, *Bromelia agavifolia* FJ968241, *Bromelia balansae* FJ968248, *Bromelia flemingii* FJ968240, *Cryptanthus bromelioides* FJ968245, *Cryptanthus dorothyae* FJ968238, *Deinacanthon urbanianum* FJ968244, *Greigia sp. RSJ 184* FJ968284, *Neoregelia cruenta* FJ968239, *Ochagavia elegans* FJ968237, *Orthophytum gurkenii* FJ968272, *Pitcairnia orchidifolia* FJ968253, *Pitcairnia sanguinea* FJ968252, *Portea fosteriana* FJ968235, *Puya alpestris* FJ968264, *Puya alpestris* FJ968269, *Puya angusta* FJ968288, *Puya angusta* FJ968290, *Puya bicolor* FJ968282, *Puya boliviensis* FJ968265, *Puya boliviensis* FJ968270, *Puya castellanosii* FJ968257, *Puya chilensis* FJ968260, *Puya chilensis* FJ968268, *Puya coerulea* FJ968263, *Puya compacta* FJ968275, *Puya dasylirioides* FJ968243, *Puya dyckioides* FJ968258, *Puya ferreyrae* FJ968289, *Puya ferruginea* FJ968250, *Puya ferruginea* FJ968285, *Puya ferruginea* FJ968286, *Puya gilmartiniae* FJ968262, *Puya goudotiana* FJ968281, *Puya harmsii* FJ968255, *Puya herrerae* FJ968287, *Puya lanata* FJ968276, *Puya lineata* FJ968283, *Puya macrura* FJ968291, *Puya mima* FJ968292, *Puya nana* FJ968254, *Puya nitida* FJ968278, *Puya nutans* FJ968266, *Puya obconica* FJ968277, *Puya pygmaea* FJ968274, *Puya raimondii* FJ968271, *Puya raimondii* FJ968293, *Puya santosii* FJ968279, *Puya trianae* FJ968280, *Puya ultima* FJ968251, *Puya venusta* FJ968261, *Puya venusta* FJ968267, *Puya wrightii* FJ968249, *Puya yakespala* FJ968259.
